# Biomimetic platelet-membrane camouflaged ivermectin nanocrystals for tumor homing and breast cancer management

**DOI:** 10.1007/s13346-025-02032-2

**Published:** 2026-01-06

**Authors:** Marwa M. Sheir, Salma E. El-Habashy, Eman Sheta, Maha M. A. Nasra, Ossama Y. Abdallah

**Affiliations:** 1 Department of Pharmaceutics, Faculty of Pharmacy, Alexandria University, 1 Khartoum Square, Azarita, P.O. Box 21521, Alexandria, Egypt; 2 Pathology Department, Faculty of Medicine, Alexandria University, Alexandria, Egypt

**Keywords:** Drug repurposing, P-selectin, Cell-membrane coating, TNBC, Metastasis, Immunotherapy

## Abstract

Leveraging the intrinsic platelet-cancer cell crosstalk through platelet membrane-coated nanosystems offers a promising avenue for targeted drug delivery, particularly against triple-negative breast cancer, the most aggressive highly metastatic breast cancer subtype. In parallel, repurposing the antiparasitic ivermectin (Ivm) for anticancer applications is hindered by poor solubility and high toxicity, restricting its parenteral administration. In this study, ivermectin nanocrystals (Ivm-NC) were first developed to enhance drug solubility and systemic delivery. Afterwards, platelet-membrane was employed for realizing platelet-mimetic-camouflaged PMV/Ivm-NC for active-targeted tumor homing, immune evasion and higher biocompatibility. The innovative PMV/Ivm-NC presented optimum particle-size and zeta-potential, while exhibiting a sustained release pattern. Successful coating and retention of platelet membrane proteins was confirmed by SDS-PAGE profiling and immunocytochemistry for the platelet-membrane-protein P-selectin. In vitro studies for PMV/Ivm-NC demonstrated higher selective-cytotoxicity (IC_50_ 2.89 ± 0.38 µg/mL) and anti-migratory potential on MDA-MB-231 cells, and cytocompatibility on normal human fibroblasts vs. uncoated Ivm-NC. In 4T1-tumor-bearing BALB/c mice, PMV-functionalization fostered preferential tumor-homing and reduced off-target distribution, compared to uncoated-NC. In addition, PMV/Ivm-NC secured pronounced tumor-growth inhibition, down-regulation of oncogenic markers (VEGF and cyclin D1), upregulation of pro-apoptotic Bax and caspase-3, and enhanced immune-infiltration of CD4^+^ and CD8^+^ T-cells, suggesting Ivm-induced immunogenic cell death. Histological evaluation confirmed higher tumor-necrosis and lower mitotic-count, as well as a notable lung-antimetastatic activity. Serum biochemistry and histopathology confirmed favorable biocompatibility. Together, our findings highlight PMV/Ivm-NC as a promising biomimetic-camouflaged nanoplatform for harnessing Ivm repurposed anticancer immunotherapy and reducing possible toxicity with selective, active targeting of triple negative breast cancer.

**Graphical Abstract:**

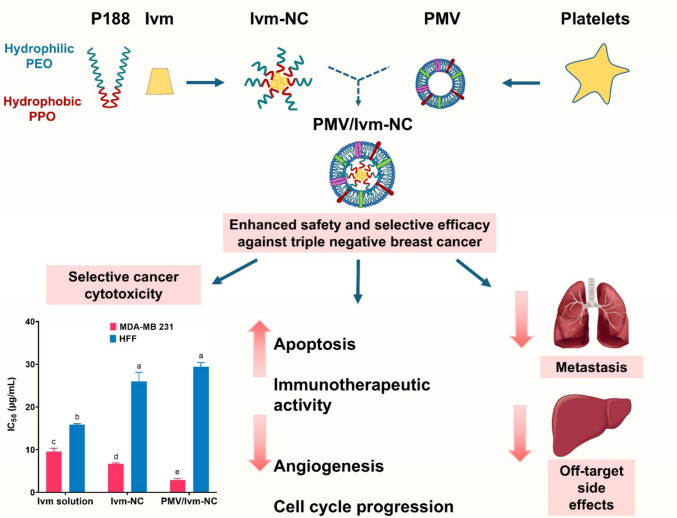

**Supplementary Information:**

The online version contains supplementary material available at 10.1007/s13346-025-02032-2.

## Introduction

Ivermectin (Ivm) is a broad spectrum antiparasitic drug derived from avermectins, a class of macrocytic lactones originally isolated from *Streptomyces avermitilis*. It was first introduced to the market in the early 1980 s for veterinary use, followed by its approval for human use against endoparasitic infections, including onchocerciasis (river blindness), filariasis, and strongyloidiasis, and ectoparasites including scabies and lice [1]. This breakthrough led to the awarding of the 2015 Nobel Prize in physiology and medicine to William C. Campbell and Satoshi Omura for their discovery and therapeutic application of avermectins [2]. In recent years, Ivm has gained growing interest for its repurposing in non-parasitic diseases including antiviral [3], anti-inflammatory [4] and anticancer properties [5, 6]. Notably, its anticancer potential has been investigated across various malignancies, where it exhibits anti-proliferative, pro-apoptotic and anti-metastatic activities through modulation of multiple cellular pathways relevant to cancer progression, such as Wnt/β-catenin signaling, PAK1, and mitochondrial dysfunction, positioning it as a promising candidate for cancer applications [6]. In particular, Ivm has been reported to exert immunogenic cell death, where a form of apoptosis promotes tumor antigen release and dendritic cell activation. This approach secures bridging chemotherapy with immunotherapy, aiming to eliminate tumor cells directly and stimulate a lasting immune response [7]. Despite these therapeutic prospects, formulation challenges remain a major obstacle owing to Ivm high lipophilicity (log P 3.2) and poor aqueous solubility (0.004 mg/mL) [8]. These shortcomings limit Ivm clinical translation for parenteral administration which is a viable route for tumor-targeted delivery, necessitating the use of co-solvents or high surfactant concentration with possible toxicity [9]. To overcome these barriers and improve Ivm delivery and therapeutic functionality, formulation of Ivm nanocrystals (Ivm-NC) as a novel carrier-free nanodrug delivery system could enhance its solubility, facilitate cellular uptake and potentiate its anticancer activity against challenging cancer types such as triple negative breast cancer management, for which Ivm has previously demonstrated an established therapeutic potential [10, 11].

Nanocrystals are pristine drug particles reduced to the nanometer scale and stabilized by surfactants or polymers to prevent aggregation. Although referred to as “drug nanocrystals,” the particles may not be entirely crystalline; depending on the preparation method, they can exhibit partial or even complete amorphous character [9]. This approach offers an increased surface-to-volume ratio, enhancing the apparent solubility and dissolution rate of poorly water-soluble drugs, thereby improving bioavailability [12]. For instance, several NC-based drug products have been successfully marketed. Examples include Emend® IV, an intravenous formulation of the antiemetic fosaprepitant; Invega Sustenna®, a long-acting intramuscular injection of paliperidone for schizophrenia; and Rapamune®, an oral sirolimus formulation initially developed using NC technology to enhance solubility and bioavailability [13]. These examples highlight the clinical feasibility and regulatory relevance of NC formulations for both oral and injectable routes [14]. When combined with surface functionalization, NC can be tailored for enhanced targeted delivery to tumor sites, including triple negative breast cancer [15].

Surface functionalization strategies have been used for imparting stealth and/or targeting properties to drug delivery systems, with cell membrane coating emerging as a revolutionary approach. Recently, advances in tumor microenvironment research have revealed complex cancer cell-host interactions, inspiring the development of cell membrane coating of nanodrug delivery systems. Such a strategy provides a versatile bioinspired approach for the realization of biomimetic and parent cell-derived drug delivery systems, with a consequently higher-level cancer cell interaction. This innovative approach exploits the various intrinsic physiological features of employed cells for camouflaging nanoparticles with parent cell membranes, enabling them to inherit the surface antigens and biological functions of the source cells, including inherent biocompatibility, cancer targeting, immune evasion and biological barrier penetration [16]. In this regard, platelet membrane coating offers unique advantages for harnessing platelet-cancer cell crosstalk. For example, platelets play an intrinsic role in vascular adhesion and interaction with solid tumor and circulating tumor cells [17]. In specific, the platelet membrane protein CD62P (P-selectin) presents a particular affinity to CD44 receptors which themselves are overexpressed in cancer cells [18]. In addition, platelets show innate homing towards circulating tumor cells (CTC) in the bloodstream, which are mainly responsible for tumor metastasis, enabling their survival from hemodynamic shear forces while ensuring immune evasion [17]. Therefore, platelet membrane-based delivery systems offer a promising approach that is particularly relevant for therapeutically challenging cancers such as the aggressive triple negative breast cancer, which lacks specific molecular targets due to the absence of estrogen receptor, progesterone receptor and human epidermal growth factor-receptor 2 expression [19]. Indeed, this cancer type is known for its aggressiveness and high relapse from a localized to metastatic state [19]. By cloaking nanocrystals with platelet-derived membranes, the resulting platelet-mimetic system can evade immune detection, prolong circulation time and exploit the innate homing ability of platelets to inflamed or tumor-associated vasculature, making them especially attractive for cancer-targeted delivery [20]. Such interaction underscores platelet membrane functionalization as an opportune approach for active targeting and tumor homing for triple negative breast cancer therapy. Moreover, leveraging platelet membrane ability to interact with and neutralize CTC offers a promising strategy for metastasis prevention [21, 22].

Although Ivm NC have been developed to improve antiparasitic efficacy [ 1, 23, 24] their application as anticancer therapy for triple negative breast cancer management remains unexplored either in vitro or in vivo. Despite the promising anticancer potential of Ivm established in both in vitro and in vivo studies, previous investigations have relied on simple solubilization approaches using oil-based vehicles [10] or solvent mixture [11] to overcome solubility challenges upon injection. However, these conventional solubilization strategies do not address the main issues of poor tumor selectivity and potential off-target toxicity. In specific, no advanced targeting strategies have been employed for Ivm delivery in breast cancer applications. Particularly, platelet-membrane coating has not yet been investigated for active-targeted tumor homing of Ivm. Therefore, there is a need to develop a tumor active-targeted Ivm drug delivery system that can harness Ivm immunotherapeutic anticancer efficacy and enhance active tumor homing while minimizing systemic exposure.

**Fig. 1:**
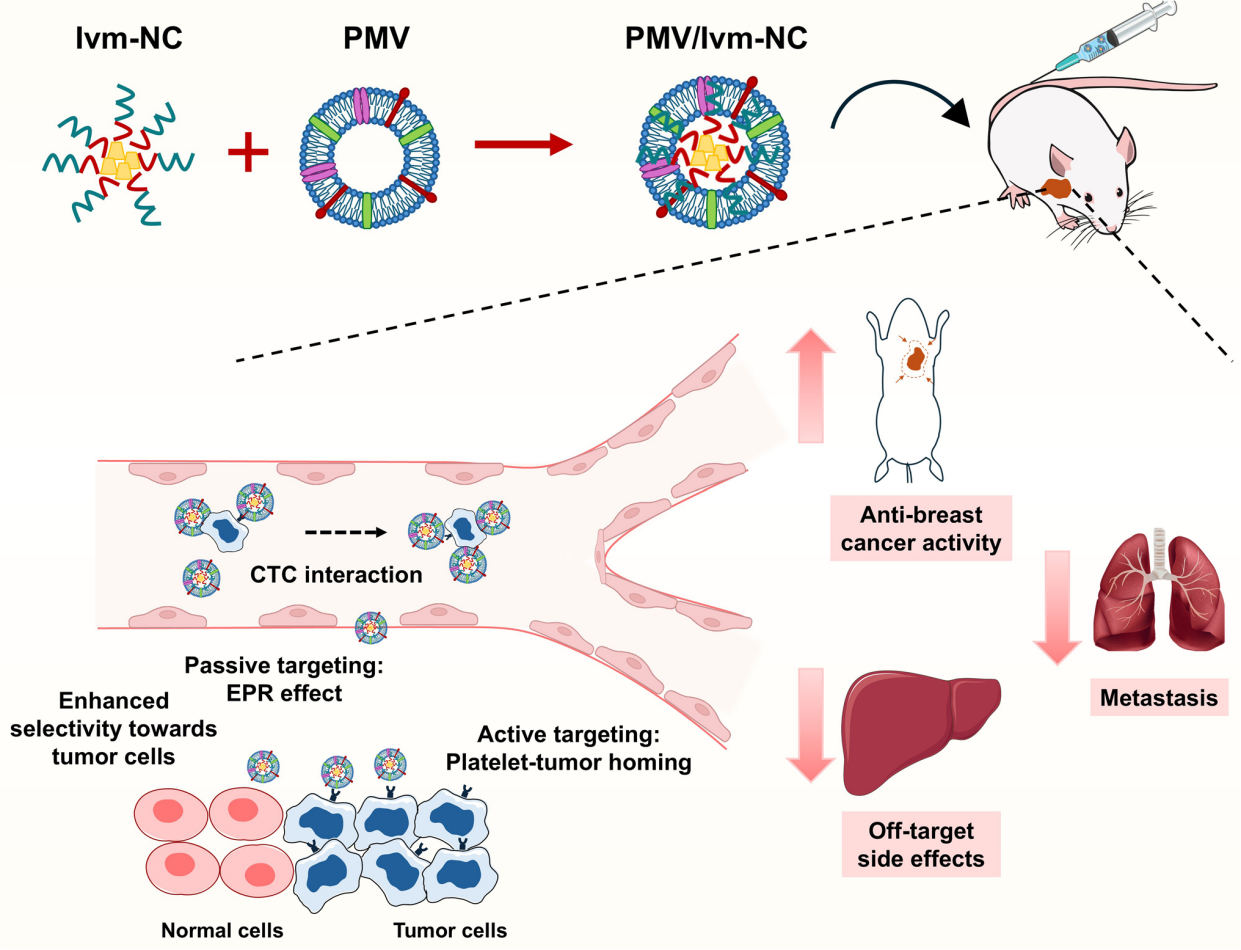
Scheme for the preparation and realization of antitumor multifunctionality of the platelet-mimetic PMV/Ivm-NC

This study aimed at leveraging Ivm anticancer, immunotherapeutic repurposing, nanocrystallization and platelet-mimetic camouflaging as a promising approach for the comprehensive management of triple negative breast cancer Fig. 1. We first prepared Ivm-NC with high drug-loading via a bottom-up sonoprecipitation method, using various stabilizers for the optimization NC yield and colloidal attributes for prospective intravenous, cancer treatment applications. Afterwards, platelet membrane was successfully isolated, with comprehensive characterization to verify membrane integrity and preserved functionality. Subsequent biomimetic platelet-membrane functionalization of Ivm-NC was performed for the realization of PMV/Ivm-NC. An exhaustive experimental toolset was conducted for the elaboration of in vitro and in vivo anticancer activity, immunotherapeutic potential and antimetastatic efficacy. In addition, hemocompatibility and biological safety were investigated.

## Materials and methods

### Materials

Ivermectin (Ivm) and poloxamer 407 (P407) were kindly gifted by Medizen Pharmaceutical Industries (Egypt). Poloxamer 188 (P188) and coumarin-6 (C6) dye were purchased from Sigma Aldrich Chemical Co. (USA). Prostaglandin E1 was obtained from Chongqing Biospes Co., Ltd, (China) and Phenylmethylsulfonyl fluoride (PMSF) was supplied form Himedia (India). Reagents for SDS-PAGE were obtained from Bio-Rad (USA). All other chemicals and solvents were of analytical grade.

### Cells

In vitro cell culture studies were performed on the human triple negative breast cancer cell line MDA-MB-231 (ATCC HTB-26™) and the non-cancerous human foreskin fibroblasts HFF (ATCC SCRC-1041). In vivo studies were conducted using murine 4T1 triple-negative breast cancer cells (ATCC CRL-2539 ™). Cells were grown in Dulbecco’s modified Eagle’s medium (DMEM)- high glucose (Biowest, France), enriched with fetal bovine serum (FBS 10% v/v, Capricorn Scientific, Germany) and antibiotics (100 U/mL penicillin and 100 µg/mL streptomycin). Cells were maintained in a 5% CO_2_ incubator at 37 °C and monitored daily for growth and morphology using a phase-contrast inverted microscope (CKX41SF; Olympus). The culture medium was refreshed every 2–3 days to ensure optimal cell viability. Experiments were conducted in the Cell Culture Laboratory, Department of Pharmaceutics, Faculty of Pharmacy, Alexandria University.

### Animals

Female BALB/c mice (6–8 weeks old, 25–30 g) were obtained from Vacsera Co., Egypt and were housed at the animal house of the Medical Research Institute, Alexandria University, Egypt. Animals were maintained at ambient temperature (25 ± 1 °C) and a 50–65% relative humidity under a 12-h light/12-h dark cycle. All animals had ad-libitum access to standard rodent chow and water throughout the study. The adopted protocols accorded with the European Parliament Directive 2010/63/EU for animal experiments and the ARRIVE guidelines. All experimental procedures were approved by the Institutional Animal Care and Use Committee (IACUC; Faculty of Pharmacy, Alexandria University; IACUC approval number; AU0620239112195).

### Preparation and optimization of ivermectin nanocrystals (Ivm-NC)

Ivermectin nanocrystals (Ivm-NC) were prepared using the sonoprecipitation method [25, 26]. Briefly, an organic solution of Ivm (0.1 or 0.2% w/v final concentration) was added to 10 mL of an aqueous solution of the stabilizer (P188 and P407) at different final concentrations (0.025% and 0.05% w/v). Mixing proceeded at 4 °C while stirring at 800 rpm for 1 min (IKA Eurostar; IKA Labortechnik, Germany). The prepared mixture was ultrasonicated at 60% amplitude and 4 °C for 10 min (Sonoplus HD 3100, Bandelin, Germany), then further stirred at 25 °C for 2 h for complete solvent evaporation [27]. The prepared nanocrystals were finally separated by centrifugation at 26,000 × g and 4 °C for 30 min (3–30 K Sigma cooling centrifuge; Laborzentrifugen GmbH, Germany).

The particle size, polydispersity index and zeta potential were measured by dynamic light scattering using Zetasizer Nano ZS (Malvern Instruments, Malvern, UK) after suitable dilution with filtered distilled water. The yield of the prepared nanocrystals was assessed using an indirect method. Briefly, following centrifugation of the prepared nanocrystal dispersion, the separated supernatant was analyzed for the quantification of free drug by UV–Vis spectrophotometry (Agilent Cary-60, USA) at λ_max_ of 245 nm [24].

For fluorescence tracking, coumarin-6 nanocrystals (C6-NC) were prepared as previously described [28], using 0.025% w/v P188 and employing similar procedure.

### Isolation and characterization of platelet-membrane vesicle (PMV)

#### Extraction of platelet-membrane vesicles (PMV)

Platelet membranes were first isolated based on previous protocols [29, 30]. Briefly, whole blood from female BALB/c mice was collected via retro-orbital venous plexus in EDTA-containing tubes (5 mM). Platelet rich plasma was isolated by centrifugation of whole blood at 200 × g and 25 °C for 20 min to separate red blood cells and white blood cells. The obtained platelet rich plasma was carefully mixed with phosphate buffered saline (PBS; pH 7.4) containing EDTA (1 mM) and prostaglandin E_1_ (2 µM) to prevent platelet activation [29]. Platelets were then pelleted by centrifugation at 800 × g and 25 °C for 20 min and dispersed in PBS containing EDTA (1 mM) and supplemented with PMSF (1 mM) [21]. Platelet membranes were then obtained by freezing the platelet dispersion at − 80 °C followed by thawing at room temperature for three freeze–thaw cycles, before separation at 12,000 × g and 4 °C for 30 min. The pelleted membranes were dispersed in deionized water. Finally, the obtained dispersion was sonicated at a frequency of 42 kHz and a power of 70 W for 10 min (Ultrasonic Branson 1510; Branson Ultrasonics Corp., USA), then extruded through a 0.2-µm polycarbonate membrane using a mini extruder (Model ER-1, Eastern Scientific LLC, USA) for 5 cycles to prepare platelet-membrane vesicles (PMV).

#### Immunocytochemical analysis of P-selectin

The expression of P-selectin (CD62P); an essential protein marker involved in platelet-cancer cell interaction, was verified by immunocytochemical analysis of PMV, compared to fresh isolated platelets. Samples were fixed with 4% paraformaldehyde for 15 min, followed by PBS washing. Non-specific binding was blocked using EnVision FLEX peroxidase blocking reagent for 5 min. After rinsing, cells were incubated with anti-P-selectin primary antibody (Biospes Co., Ltd; 1:100 dilution) for 30 min. Subsequently, the EnVision FLEX/HRP polymer was applied for 20 min, and antibody binding was visualized using EnVision FLEX DAB + chromogen diluted in substrate buffer for 10 min. Slides were then examined under a light microscope (Olympus BX41, Japan) [31].

### Formulation and optimization of biomimetic platelet-membrane vesicle (PMV) camouflaged ivermectin nanocrystals (PMV/Ivm-NC)

Functionalization of the developed nanocrystals with PMV was carried out by co-sonication [32]. Briefly, the prepared Ivm-NC were mixed with PMV dispersion at different drug/protein ratios [33] (1:0.025, 1:0.05 and 1:0.1 w/w), where total protein content was quantified by Bradford’s assay [32, 34]. The mixture was then sonicated at 70 W power for 10 min before further use. Colloidal properties were investigated for all the developed biomimetic PMV-camouflaged NC. The selected formulation was additionally assessed after 1 week of storage at 4^◦^C.

### Microscopic examination

#### Transmission electron microscopy (TEM)

The ultrastructural morphology of different samples was examined using TEM (Jeol, JEM-100 CX, Japan). Samples were appropriately diluted and stained with 2% uranyl acetate. A drop of sample was deposited onto a carbon-coated grid and allowed to dry before imaging.

#### Scanning electron microscopy (SEM)

The surface morphology of different samples was examined using SEM (SM-IT200; JEOL, Japan). Samples were mounted on metal stubs, air-dried and sputter-coated with gold before imaging.

### Whole protein profiling

Total protein expression was analyzed for PMV and PMV/Ivm-NC compared to fresh platelets via SDS-PAGE for protein separation followed by Coomassie brilliant blue staining for visualization, as previously described [35]. Briefly, samples were heated at 95 °C for 5 min, loaded onto an SDS–polyacrylamide gel (8–12% w/v), together with a protein marker, and run at 120 V for 1 h. The resulting gel was stained with Coomassie blue, followed by decolorization and imaging of the protein bands with a digital camera. All the samples were normalized to equivalent protein concentration using Bradford’s assay [32, 34].

### Fourier transform infrared spectroscopy (FTIR)

FTIR spectroscopy (Agilent Cary 630; Agilent Technologies, USA) was performed to evaluate potential interactions among the components of the prepared systems. Lyophilized samples (LyoQuest, Telstar, Spain) were investigated over the range of 4000 to 650 cm^−1^.

### Powder X-ray diffraction (XRD)

The crystalline behavior of the prepared NC was investigated using XRD analysis (XRD-7000, Bruker D2-Phaser, Germany) [36]. The diffraction patterns were recorded in step scan mode at 40 kV and 40 mA. Measurements were conducted over a 2θ-range from 5° to 80° at 0.02°- step size.

### In vitro drug dissolution study

In vitro drug dissolution profile was evaluated for Ivm-NC and PMV/Ivm-NC compared to both drug solution (in methanol) and coarse drug suspension (in deionized water) using the dialysis method [25, 37]. Samples corresponding to 1 mg drug were placed within dialysis bags (Visking®, MWCO 12,000–14,000; SERVA, Germany) and immersed in 15 mL of the release medium (40% v/v methanol in PBS, pH 7.4), ensuring sink conditions, as determined by preliminary solubility testing. Experiments were run at 37 ± 0.5 °C under constant shaking of 100 rpm (Wisebath, Daihan Scientific Co. Ltd, South Korea). At predetermined time points, samples were aliquoted and fresh medium was added, then drug spectrophotometric quantification was performed at λ_max_ 245 nm.

### In vitro anticancer cell studies

#### In vitro cytotoxicity by MTT assay

The in vitro cytotoxic effects of different NC formulations were tested on MDA-MB 231 cells using the MTT assay [38]. Briefly, cells were seeded at a density of 7 × 10^3^ cells/well in 96-well plates (Corning Costar Corp., USA) and cultured for 24 h. The cells were then incubated with culture medium (control) or different treatments; Ivm solution or different NC formulations (at 0.78–25 µg/mL drug concentration). After 48 h, the treatment was removed, and cells were incubated with MTT solution (1 mg/mL) for 4 h. Afterward, DMSO was added to dissolve the formazan crystals, and the absorbance was measured at 570 nm using a multiplate reader (ELX 800; Biotek, USA). The viability values of untreated cells served as the 100% viability control. Dose–response curves were plotted and IC_50_ values were calculated accordingly.

In vitro cytocompatibility was similarly performed on human foreskin fibroblasts (HFF), as normal, non-cancerous cells. A selectivity index was then calculated as the HFF IC_50_/MDA-MB 231 IC_50_ value [39].

#### Intracellular uptake

The intracellular uptake and affinity of the developed NC formulations to MDA-MB-231 cells was investigated employing C6-NC [28]. Briefly, coverslips were placed in 6-well plates, then cells were seeded at 2 × 10^5^ cells/well and incubated for 24 h. Cells were then treated with C6 solution, C6-NC or PMV/C6-NC (at a final C6 concentration of 0.1 µg/mL). At 4 h and 24 h post-treatment, culture media were removed, and cells were PBS-washed, fixed with 4%-paraformaldehyde for 20 min and washed again with PBS. Nuclei were stained with Hoechst 33342 and fluorescence imaging was performed using confocal microscopy (Model DMi8, Leica® Microsystems Inc., Germany) at an excitation wavelength of 355 nm. Intracellular fluorescence intensity, indicating cellular uptake, was quantified using image analysis (Fiji; National Institutes of Health, USA) [36] of at least 4 different fields.

#### Cell migration assay

The migration ability of MDA-MB 231 cells treated with different NC formulations was evaluated using a wound healing assay [38]. Cells were seeded in a 12-well plate at a density of 2 × 10^5^ cells/well and incubated overnight to allow the formation of a confluent monolayer. A uniform scratch was created in the cell monolayer in each well with a sterile pipette tip. Cells were then treated with Ivm solution or different NC formulations at concentrations corresponding to half their IC_50_ values for 24 h. Scratch closure was monitored by imaging of the same regions at 4 h and 24 h post-treatment. The captured images were analyzed using imaging analysis of at least 4 different fields and the percentage of wound closure was calculated using Eq.1:

1 $$\begin{array}{c}Wound\;closure\;(\%)\;=\frac{\mathrm{Initial\;wound\;area}\;-\;\mathrm{Remaining\;wound\;area}}{\mathrm{Initial\;wound\;area}}\;\times\;100\end{array}$$

#### Apoptosis assay (Annexin V-FITC/Propidium iodide assay)

The proapoptotic effect of different NC formulations on MDA-MB 231 cells was investigated using the Annexin V assay. Cells were seeded at a density of 2 × 10^5^ cells/well in a 6 well plate and incubated for 24 h. Cells were then treated for 24 h with Ivm solution, Ivm-NC and PMV/Ivm-NC at concentrations equivalent to half their IC_50_ values. Afterwards, cells were trypsinized, collected by centrifugation and stained with annexin V-fluorescein isothiocyanate (FITC) and propidium iodide (PI) as per the manufacturer’s protocol. Analysis of apoptotic cells was performed by flow cytometer (BD FACSCalibur™ flow cytometer, USA).

### Hemocompatibility studies

The hemocompatibility of different NC formulations was assessed by testing their in-vitro RBCs hemolytic activity, as previously reported protocols [36]. Briefly, fresh blood was collected from healthy mice in heparinized tubes. RBCs were sedimented by centrifugation (2000 rpm, 15 min), washed twice with normal saline (0.9% w/v) and dispersed in normal saline to obtain 2% v/v concentration. The RBCs suspension was then incubated with the tested NC formulations (at 1:1 v/v ratio) at 100 rpm and 37*°*C for 1 h. RBCs incubated with 1% v/v Triton-X 100 and normal saline served as positive control (100% hemolysis) and negative control (0% hemolysis), respectively. RBCs were then pelleted by centrifugation at 3000 rpm for 5 min, and hemolyzed hemoglobin in the supernatant was quantified by spectrophotometric analysis at λ_max_ 540 nm. Percentage hemolysis was quantified as per Eq.2:

2 $$Hemolysis\;(\%)\;=\frac{\mathrm{Absorbance\;of\;sample}\;-\;\mathrm{Absorbance\;of\;negative\;control}}{\mathrm{Absorbance\;of\;positive\;control}\;-\;\mathrm{Absorbance\;of\;negative\;control}}\;\times\;100$$

The in vivo tail bleeding assay was performed to evaluate the effect of the different treatments on the bleeding time [22]. Twelve BALB/c mice were divided into three groups (n = 3), receiving intravenous (IV) administration of either saline (control), Ivm-NC or PMV/Ivm-NC (at 6 mg/kg Ivm concentration) every other day for 14 days [10, 11]. Afterwards, a 5 mm section was excised from the distal end of the tail using a sterile scalpel, and the tail was immediately submerged in a 50 mL tube of phosphate-buffered saline (PBS) at 37 °C. The time taken for bleeding to cease was recorded for each mouse and was denoted as bleeding time.

### In vivo biodistribution

The biodistribution and tumor-targeting efficiency of the NC formulations were assessed in BALB/c mice bearing orthotopic 4T1 breast tumors, as previously described [40], employing C6 as fluorescent probe. Twelve mice were used, where an orthotopic tumor was induced via fat pad inoculation of 4T1 cells (2 × 10^6^ cells in 100 µL PBS) at the second mammary gland of each mouse. After tumor volume reached a palpable mass, tumor-bearing mice were divided at random into 3 groups (n = 4), receiving a single IV dose of C6-NC, or PMV/C6-NC (equivalent to 500 µg/kg coumarin) via the tail vein. A control group (receiving saline) was also assigned. After 6 h, the mice were sacrificed, tumors and major organs (lungs, liver and kidneys) were harvested, fixed in 10% v/v formalin, and embedded in paraffin. Tissue sections were imaged by fluorescent microscopy (Olympus BX41, Japan), and coumarin-6 fluorescence intensity was quantified using image analysis of at least 4 different fields.

### In-vivo anticancer studies

#### Induction of orthotopic 4T1 breast cancer model

The anticancer activity of the prepared NC formulations was evaluated using an orthotopic 4T1 triple negative breast cancer murine model, as previously described [11]. Twenty BALB/c mice were employed for tumor induction by injection of 2 × 10^6^ 4T1 cells into the fat pad of the second mammary gland. Tumor growth was monitored by measuring the longest (length) and shortest perpendicular (width) diameters using a vernier caliper. Tumor volumes were then estimated using **Eq. **3:

3 $$Tum\;or \;volume=0.5\times Length\times {Width}^{2}$$

#### Experimental design and treatment groups

After the development of a palpable tumor mass, animals were randomly assigned into 4 groups (n = 5). Treatment groups received saline (untreated, positive control), Ivm solution (in 60% PEG 400 in 0.9% saline) [41], Ivm-NC or PMV/Ivm-NC (equivalent to 6 mg/kg Ivm [10]) via IV administration. Healthy mice (negative control) group receiving normal saline was also assigned. Treatment proceeded at 3 doses/week for 2 weeks. Afterwards, mice were euthanized by cervical dislocation under isoflurane anesthesia [42]. Tumor tissues and different organs were carefully weighed and collected for further investigation.

#### Evaluation of anti-tumor activity

##### Evaluation of tumor growth

Animal weights and tumor growth were monitored throughout the study, where tumor volumes were recorded for all groups three times per week. The percentage change in tumor volume was determined by normalizing the measured tumor volume at each time point to its initial volume. Body weight and general behavior of the mice were also monitored to evaluate potential toxicities.

##### Quantitative real time polymerase chain reaction (qRT-PCR)

The expression of selected tumor-associated genes was investigated by qRT-PCR. The relative expression of apoptosis-related genes: Caspase-3 (F: AGGGGTCATTTATGGGACA and R: TACACGGGATCTGTTTCTTTG) and Bcl-2 Associated X-protein (BAX; F: GCTGACATGTTTGCTGATGG and R: GATCAGCTCGGGCACTTTAG) was quantified. The housekeeping gene GAPDH (F: AGCTTGTCATCAACGGGAAG and R: TTTGATGTTAGTGGGGTCTCG) was used for normalization. The total RNA was isolated from excised tumor tissues (ABT RNA extraction kit; Applied Biotechnology, Egypt) and quantified (NanoDrop DS-11 FX, DeNovix, USA). Reverse transcription was performed (ABT 2X RT mix; Applied Biotechnology, Egypt), then qRT-PCR was run using a SYBR Green Master Mix (ABT 2X qPCR Mix, Applied Biotechnology, Egypt) on QuantStudio 1 Real-Time PCR System (Applied Biosystems; Thermo Scientific, USA). Relative gene expression was determined using the comparative threshold cycle (2^−ΔΔCt^) method, normalized to GAPDH and untreated controls.

##### Enzyme Linked Immunosorbent Assay (ELISA)

Tumor growth biomarkers were determined using enzyme-linked immunosorbent assay (ELISA), as previously described [39]. Following sacrifice, extracted tumor samples were weighed and homogenized in PBS at a final concentration of 10% tissue homogenate. The expression levels of vascular endothelial growth factor (VEGF; CUSABIO, China) and cyclin D1 (Mouse CCND1 Cyclin D1 ELISA Kit, ELK Biotechnology CO., LTD, China) were quantified as indicators of angiogenesis and cell cycle progression, respectively. Total protein content in tissue lysates was quantified using Bradford assay. Levels of VEGF and cyclin D1 were normalized to total protein [43].

##### Histopathological, histomorphometric and immunohistochemical analysis

Excised tumors were fixed immediately in 10% neutral-buffered formalin. Tissues were processed and embedded in paraffin. Using semi-automated rotatory microtome, paraffin blocks were sectioned into 5-µm-thick slices. Sections were stained with hematoxylin and eosin (H&E) and examined using light microscopy. At × 100 power, tumors were photographed to assess areas of necrosis. They were seen as eosinophilic structureless granular areas. Then at high power (× 400), cells were assessed for viability, inflammatory cells infiltration and mitotic activity. Mitotic figures were counted at 10 hot spot areas then the mean count/HPF (high power field) was calculated [39].

For the assessment of tumor infiltrating CD4^+^ and CD8^+^ T lymphocytes, immunohistochemical staining of tumors was performed. Tumor sections were deparaffinized, rehydrated and immunostained using link48 Dako autostainer. Primary antibody specific to CD4 (#IR649, mouse monoclonal antibody, clone 4B12, ready to use) and CD8 (#IR623, mouse monoclonal antibody, clone C8/144B, ready to use) were used. After washing, sections were incubated with HRP-conjugated secondary antibodies and developed using a DAB substrate kit. Counterstaining was performed with hematoxylin, and slides were imaged using a light microscope (Olympus, CX-23, Japan). The count of CD4^+^ or CD8^+^ cells was done per HPF. Positive lymphocytes were counted manually using Fiji software avoiding any nonspecific stain in necrotic areas or in stroma.

##### In vivo toxicity study

Liver, spleen and kidney were excised from sacrificed animals and fixed in 10% formalin. Tissues were processed into paraffin blocks. Five micron-thick sections were cut and stained by H&E to assess any pathologic effect of studied drugs.

##### Biochemical blood analysis

Blood was collected at the end of the study for biochemical analysis. Serum was separated and stored at −  20 °C until processing. Samples were analyzed for liver enzymes (ALT; alanine transaminase, AST; aspartate transaminase and ALP; alkaline phosphatase) and kidney functions (urea, creatinine) [36].

### In vivo* anti metastasis study*

A breast cancer-lung metastasis model was established via tail vein intravenous injection of 4T1 cancer cells in BALB/c mice, as previously described [22, 44, 45]. Female BALB/c mice were injected with 4T1 cells (3 × 10^5^ cells in 100 µL PBS) via tail vein injection. Treatment was initiated 30 min post-injection of cancer cells by intravenous injection of Ivm solution, Ivm-NC and PMV/Ivm-NC (corresponding to 6 mg/kg Ivm) compared to control saline. Afterwards, treatment continued 3 times per week for 2 weeks. At the end of the study, mice were sacrificed, and lungs were excised and processed into paraffin blocks. H&E-stained sections were assessed under light microscope for the presence or absence of metastatic tumor deposits.

### Statistical analysis

All experiments were performed in triplicate, and results are expressed as mean ± standard deviation. Statistical analysis was conducted using analysis of variance (ANOVA) with Tukey’s post hoc test in SPSS 20® (SPSS Inc., USA), with significance set at *p* ≤ 0.05.

## Results and discussion

### Formulation and optimization of ivermectin nanocrystals (Ivm-NC)

In this study, Ivm-NC were innovatively prepared for the repurposed immunotherapeutic triple negative breast cancer management. Previously, Ivm has been formulated as NC via a top-down homogenization approach [24], a microfluidization technique [23] and antisolvent recrystallization [1]. However, the employment of sonoprecipitation method for the optimization of Ivm-NC has not been tested to date. Ultrasonic treatment has been reported to improve the stability of NC by improving the mixing efficiency, reorganizing surfactant molecules at the solid–liquid interface, hence reducing particle size, preventing aggregation and improving the homogeneity of particle size distribution [46]. In this regard, poloxamers are biocompatible and generally-regarded-as-safe non-ionic amphiphilic copolymers that provide essential surface activity as well as steric stabilization to nanoparticles, preventing their aggregation in colloidal systems [14]. In our study, we investigated the effect of using two different poloxamer stabilizers (P188 and P407) at different final concentrations (0.025% and 0.05% w/v) at 0.1% w/v Ivm on the colloidal properties and process yield of Ivm-NC formulated using sonoprecipitation method. Constant process parameters were selected based on preliminary tests and previous reports [25, 27], including sonication time (10 min) and amplitude (60%).

**Fig. 2:**
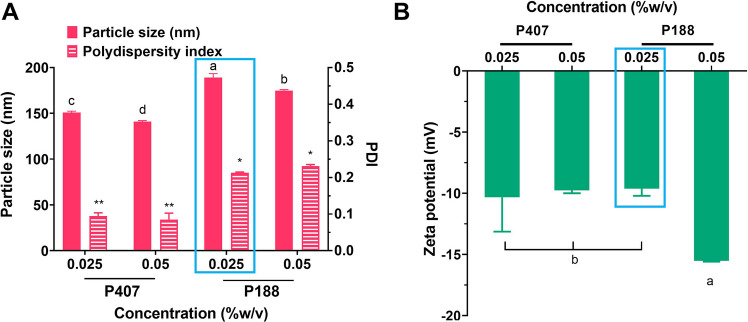
Formulation and optimization of Ivermectin nanocrystals using different stabilizers at different concentrations (**A** and **B**). Colloidal properties (**A**) and zeta potential measurement (**B**) of the developed nanocrystals. Rectangular frames outline the selected Ivm-NC formulation. Data represents mean ± SD, *n* = 3. Letters and symbols indicate statistically significant difference at *p* ≤ 0.05: a > b > c > d, * > **

The obtained particle size values for all the developed NC formulations were favorably within the desirable nano range of ≤ 200 nm (Fig. 2A**)**, which is optimal for exploiting the enhanced permeation and retention (EPR) effect in tumor targeting [47] while decreasing the possibility of clearance by mononuclear phagocyte system [48]. In addition, homogenous particle size distribution (PDI ~ 0.2) was obtained, which is essential for consistent drug delivery [47]. These results verify the opportune convenience of the sonoprecipitation technique for Ivm-NC optimization. For both stabilizers, an increase in concentration from 0.025% to 0.05% w/v was associated with a significant (*p* ≤ 0.05) decrease in particle size with no notable change in PDI values (Fig. 2A**)**. Indeed, increasing the stabilizer concentration results in more homogenous surface coverage, providing higher steric repulsion between individual NC and limiting possible crystal growth [46]. Interestingly, NC formulated using P407 demonstrated lower (*p* ≤ 0.05) particle size values (150.9 ± 1.5 and 141.0 ± 1.0 nm) compared to P188 (189.0 ± 4.4 and 174.8 ± 1.2 nm) at 0.025% and 0.05% w/v, respectively. The same pattern was mirrored for PDI values for both stabilizers. The smaller particle size and PDI values observed with P407 may be attributed to its higher hydrophobic polypropylene oxide (PPO) content, which enhances its adsorption onto the hydrophobic drug surface, resulting in more effective steric stabilization and inhibition of crystal growth compared to P188 [49, 50]. Similar findings were reported by Zhao et al. where P407 successfully stabilized tanshinone nanocrystals using a precipitation-high-pressure homogenization method. Tanshinone formulation achieved a PS of 315.7 nm and a PDI of 0.2 with 1% P407, whereas P188 failed to produce nanocrystals and resulted in microparticles.

The zeta potential for all the prepared formulations presented a reasonable magnitude negative surface charge (Fig. 2B**)**, prospectively suggesting high colloidal stability. A specific increase in zeta potential (*p* ≤ 0.05) was only noted for P188 at 0.05% w/v (−15.5 ± 0.01 mV) compared to other NC formulations (average of −9.9 mV). Negative zeta potential values are commonly reported for both P188 and P407- stabilized drug nanocrystal [51–53].

The process yield for all the NC prepared using P188 (average 89.7%) was significantly (*p* ≤ 0.05) higher than P407 (average 74.6%), with no difference between both concentrations (0.025 and 0.05% w/v), establishing high prospective scalability and cost-effectiveness. This finding can be interpreted in light of the much lower critical micelle concentration of P407 (0.03 mg/mL) compared to P188 (4 mg/mL) [48], which might have contributed higher solubilization of hydrophobic drug molecules by P407 at the same stabilizer concentration, and hence lower NC process yield was obtained for P407 compared to P188. It should be noted that NC prepared at 0.2% w/v Ivm using both stabilizers at 0.05% w/v concentration resulted in obvious precipitation on standing for 12 h and hence were exempted from further investigations.

Given these results, the NC formulation prepared using P188 at 0.025%w/v demonstrated optimum nanosize (189 nm), PDI (0.21) and zeta potential (−9.9 mV) at a high process yield (92.7%) and was therefore selected for further studies. It was henceforth denoted as Ivm-NC.

### Optimization and characterization of biomimetic PMV-camouflaged Ivm-NC (PMV/Ivm-NC)

**Fig. 3:**
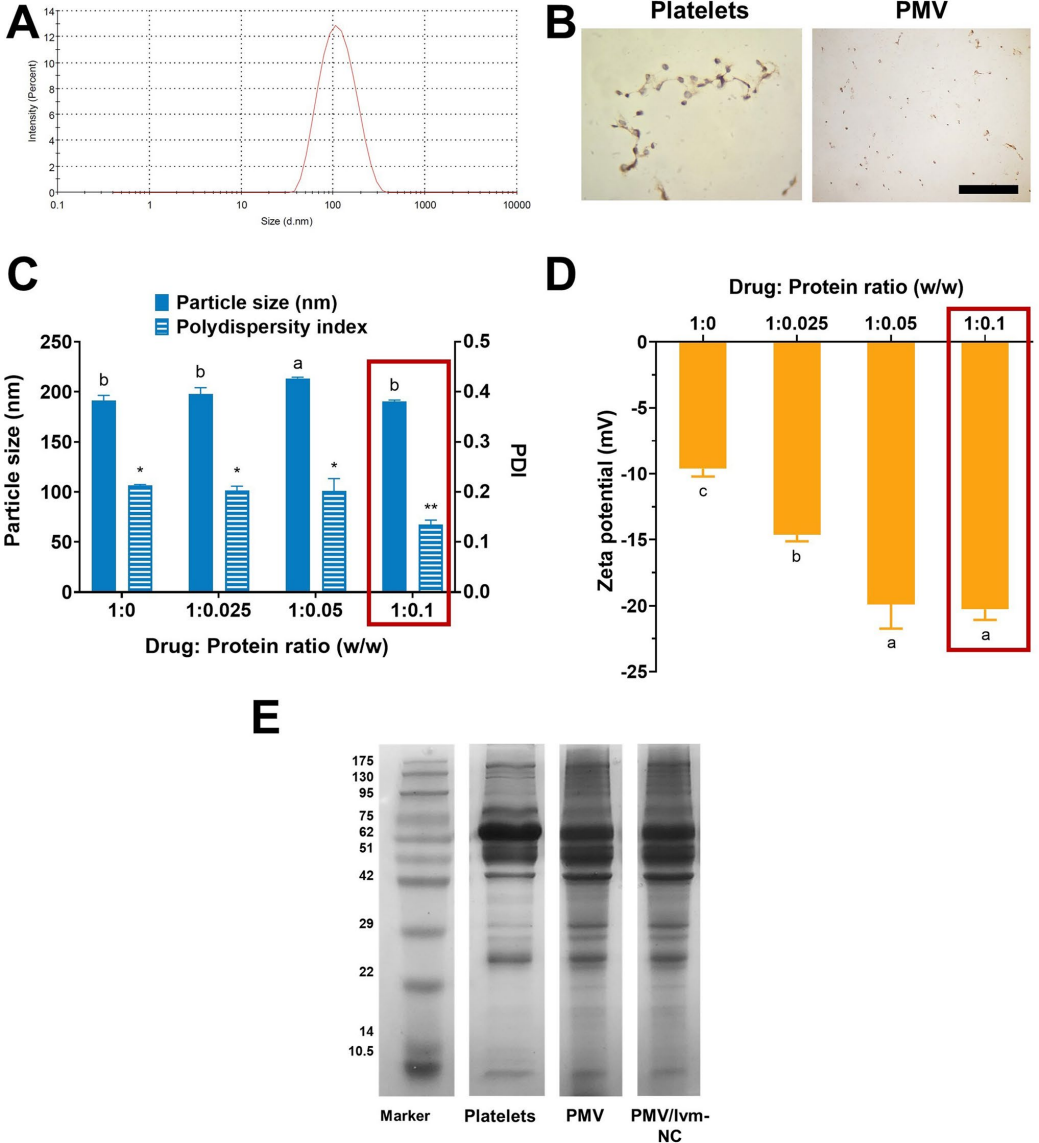
Optimization of platelet-membrane vesicle (PMV) camouflaged ivermectin nanocrystals (PMV/Ivm-NC) (**A**–**E**). Particle size (**A**) and immunocytochemical analysis of CD62P (P-selectin) expression for the isolated PMV(**B**), scale bar = 100 µm. Colloidal properties (**C**) and zeta potential measurement (**D**). Rectangular frames outline the selected PMV/Ivm-NC formulation. SDS-PAGE and Coomassie blue staining (**E**) for whole protein profiling for the optimization of PMV/Ivm-NC. Data represents mean ± SD, *n* = 3. Letters and symbols indicate a statistically significant difference at *p* ≤ 0.05: a > b > c and * > **

Platelet membranes were utilized to create a biomimetic camouflage and functional coating of the developed NC, leveraging platelet-cancer cell crosstalk and native platelet surface proteins to promote immune evasion and enhance cancer cell targetability through specific surface protein interaction [54]. Specifically, CD62P (P-selectin) is an endothelial cell adhesion molecule and an activated-platelet protein with established affinity/binding to the CD44 receptor, overexpressed on cancer cells [30]. We first isolated platelets from whole blood of BALB/c mice by centrifugation, then platelet membranes were obtained by a freeze–thaw technique. Platelet membrane vesicles (PMV) were finally derived by sonication and extrusion. The isolated PMV were characterized for colloidal properties, and the expression of P-selectin was immunocytochemically verified. The obtained PMV nanovesicles presented a nano particle size (104.3 ± 1.7 nm; Fig. 3A), uniform distribution (PDI of 0.18 ± 0.01) and a negative surface charge (zeta potential of −19.9 ± 1.49 mV). Additionally, the expression of CD62P (P-selectin) on isolated platelets was effectively inherited by the nanovesicular PMV** (**Fig. 3B), verifying favorable membrane protein retention and reliable processing parameters for PMV isolation.

For the subsequent realization of PMV-camouflaged Ivm-NC, co-sonication was employed at different drug/protein ratios (1:0.025 to 1:0.1 w/w) for the optimization of colloidal properties. Results indicated there was no change in particle size for PMV-camouflaged NC at both 1:0.025 and 1:0.1 w/w drug/protein ratio (average 194 nm; Fig. 3C**)**, compared to uncoated Ivm-NC. However, a significant (*p* ≤ 0.05) increase in particle size (213.4 ± 1.19 nm) was noted for 1:0.05 w/w ratio. Functionalization at drug/protein ratio 1:0.1 w/w resulted in the lowest (*p* ≤ 0.05) PDI value (0.13 ± 0.01), suggesting improved colloidal stability and uniformity. The zeta potential measurements (Fig. 3D**)** reflected a significant (*p* ≤ 0.05) increase in the values of negative charge on increasing drug/protein ratio for 1:0.025 (− 14.63 ± 0.49 mV) and 1:0.05 w/w (− 19.60 ± 1.83 mV), compared to uncoated Ivm-NC (− 9.62 ± 0.59 mV). Interestingly, there was no further change in surface charge for drug/protein ratio 1:0.1 w/w (− 20.26 ± 0.81 mV), which was closely similar to the zeta potential of isolated PMV (− 19.90 ± 1.49 mV), suggesting saturation of the coating process and verifying successful PMV translocation onto Ivm-NC and surface coating. Further increasing the coating ratio was considered unnecessary, since zeta potential plateau was reached and the highest coating ratio remained compatible with PMV yield and ethical constraints. Previous reports adopted similar lipid-to-membrane protein (1:0.005–1:0.05) for PMV functionalization of nanoparticles [55].

It is possible that increasing drug/protein ratio from 1:0.025 to 1:0.05 was associated with uneven PMV deposition and bridging flocculation of surface coating [56]. Whereas a further increase in drug/protein ratio to 1:0.1 resulted in more homogenous PMV surface coverage, optimum surface charge, less particle aggregation and hence lower and more uniform particle size.

Based on these findings, PMV functionalization at drug/protein ratio 1:0.1 w/w presented optimum particle size (190.2 ± 1.6 nm), PDI (0.134 ± 0.009) and zeta potential value (− 20.26 ± 0.80 mV), similar to the extracted PMV. Notably, after 1 week of storage at 4 °C, the formulation retained its colloidal properties, with particle size (182.7 ± 3.9 nm), PDI (0.271 ± 0.01), and zeta potential (− 18.5 ± 0.81 mV) remaining within acceptable ranges. As such, optimum colloidal properties are favorably established at maximum PMV content for prospective passive (via EPR effect) and active targeted, immunotherapeutic anticancer functionality. Therefore, the drug/protein ratio 1:0.1 w/w was selected as optimum and further employed for the innovative, biomimetic PMV functionalization of Ivm-NC, assigned as PMV/Ivm-NC.

### Validation of membrane protein retention

For the verification of whole protein profile of the developed PMV/Ivm-NC, SDS-PAGE followed by Coomassie blue staining was performed. SDS-PAGE analysis (Fig. 3E**)** revealed the protein bands corresponding to the original parent platelets, the isolated PMV and biomimetic camouflaged PMV/Ivm-NC were consistent. These findings indicate successful retention and efficient inheritance of key membrane proteins throughout the isolation and functionalization process, which highlights the promising prospects of PMV/Ivm-NC for the targeted anticancer functionality.

### Morphological examination

**Fig. 4:**
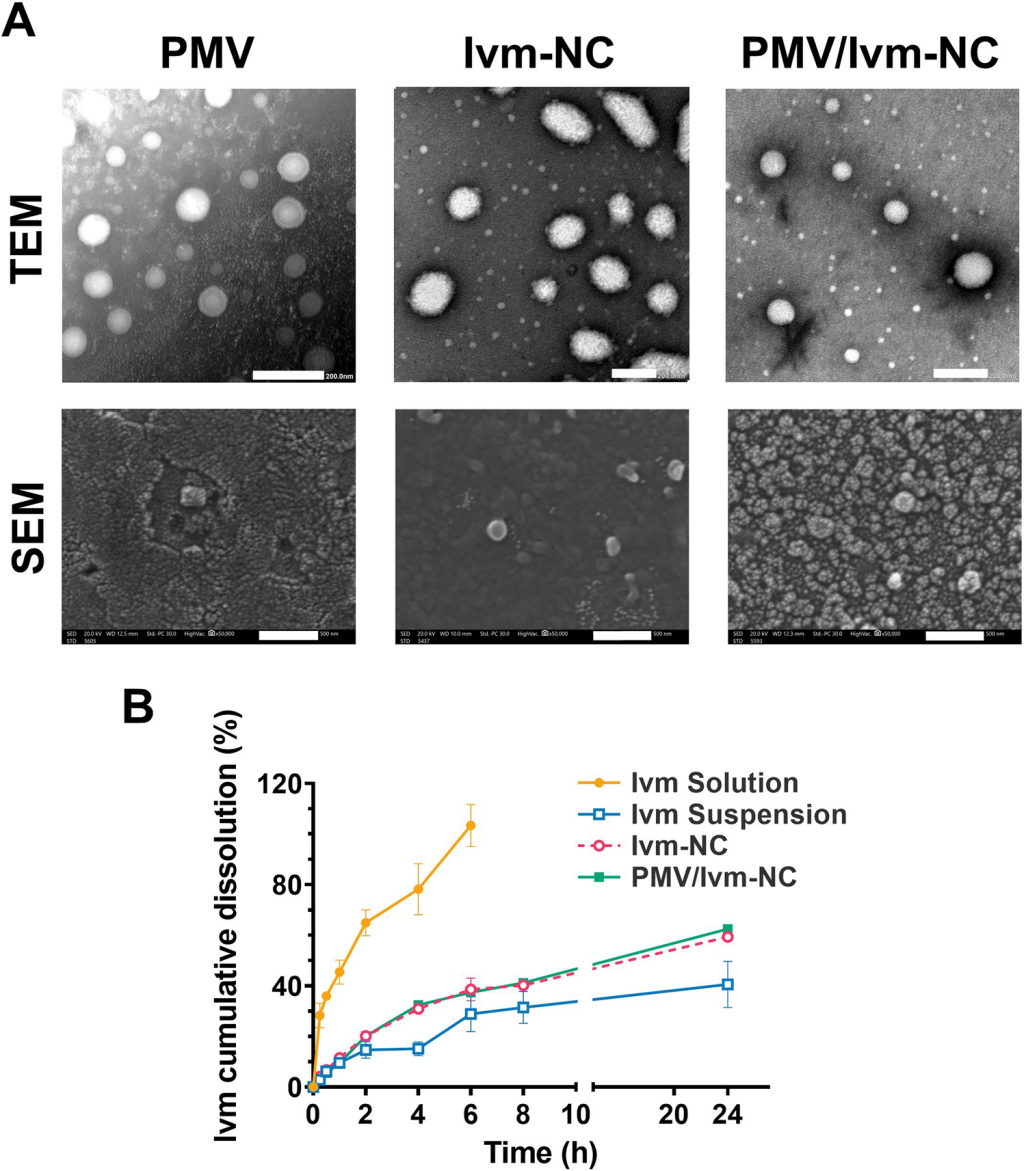
Characterization of the developed nanocrystals (**A** and **B**). Microscopic examination of the developed nanocrystals employing TEM (upper panel) and SEM (lower panel) **(A**). In vitro drug dissolution (**B**), at 37 °C and 100 rpm. Data represents mean ± SD, *n* = 3

Morphological features of the developed formulations were investigated via TEM and SEM examinations. The TEM images (upper panel; Fig. 4A) of the isolated PMV demonstrated a typical vesicular morphology, which is indicative of their lipid bilayer structure, and is consistent with previous observations of isolated membrane vesicles [32]. Both Ivm-NC and PMV/Ivm-NC showed a homogenous nanosize range, spherical morphology, which aligns with dynamic light scattering particle size measurements. A darker, surface corona could be noted for PMV/Ivm-NC, which might be illustrative of PMV surface deposition. In addition, uncoated Ivm-NC displayed a relative roughness at particle border corresponding to NC surface, which disappeared into a smoother border after PMV functionalization, similar to previous findings [57].

The SEM images (lower panel; Fig. 4A) of uncoated Ivm-NC showed spherical particles of homogenous surface. In contrast, PMV/Ivm-NC exhibited a more rough-textured surface, reflective of successful deposition of PMV on the surface of Ivm-NC, similar to a previous report [58].

### Fourier transform infrared spectroscopy (FTIR)

Functional group analysis was performed via FTIR for the elucidation of possible interactions between Ivm and P188 in Ivm-NC compared to an ingredient physical mixture.

As illustrated in Figure S1(A), the FTIR spectrum of Ivm exhibited characteristic peaks corresponding to its functional groups, including O–H stretch around 3485 cm⁻1, C − H stretching around 2960 cm-1 and C = O stretch at 1729 cm⁻1 [59]. The P188 spectrum presented distinct bands for aliphatic C − H stretch at 2878 cm^−1^, and asymmetric C − O stretch at 1098 cm^−1^ [60]. The FTIR spectrum of Ivm-NC revealed no significant shift in the characteristic peaks of Ivm, suggesting the absence of chemical interactions and confirming the compatibility of Ivm with P188. In accordance, the physical mixture displayed a simple superimposition of both individual spectra, indicating no significant chemical interaction upon simple mixing.

### Powder X-ray diffraction (XRD)

The XRD patterns of raw Ivm, P188, their physical mixture and Ivm-NC are illustrated in Figure S1(B)**.** The diffraction pattern of raw Ivm demonstrated a sharp distinct peak at 2θ around 9.5° and lower intensity peaks at 12.5°, 13.3° and 14.8°, confirming the crystalline nature of the raw drug [23]. Likewise, the pattern of P188 showed two distinct peaks at 2θ around 18.8° and 22.9°, characteristic of its semicrystalline structure, where P188 comprises semicrystalline polyethylene oxide segments and amorphous polypropylene oxide segments [61]. Peaks of raw Ivm and P188 were collectively present in their physical mixture, with varying intensities. Interestingly, the XRD spectrum of Ivm-NC demonstrated broad humped shoulders of low intensity and no characteristic peaks for Ivm or P188. This pattern suggests the amorphization of the P188-stabilized nanosized drug as NC. This finding is consistent with previous reports on the formation of SDS-stabilized Ivm-NC prepared by microfluidization [23] and polyvinylpyrrolidone-stabilized Ivm-NC by high pressure homogenization [24].

### In vitro drug dissolution

The in vitro dissolution was investigated for PMV/Ivm-NC and Ivm-NC compared to both Ivm solution (in methanol) and coarse suspension (in deionized water). Drug solubility was screened prior to the dissolution study, where 40% v/v methanol in PBS could adequately solubilize Ivm (340 µg/mL) and was hence selected as optimum release medium.

The in vitro dissolution profiles (Fig. 4B**)** demonstrate the rapid and complete diffusion of Ivm solution (100 ± 8.32% within 6 h), confirming effective drug dialyzability in the selected release medium. In contrast, Ivm coarse suspension expectedly presented low extent and slow drug dissolution, with only 28.86 ± 7% and 40.54 ± 9.15% drug dissolved after 6 h and 24 h, respectively. This pattern could be attributed to the poor aqueous solubility and limited dissolution and diffusion of raw Ivm. Interestingly, both Ivm-NC and PMV/Ivm-NC similarly afforded controlled Ivm dissolution in a steadily increasing pattern, with ~ 60% of the drug released by 24 h, suggesting no substantial impact of PMV functionalization on drug diffusion [62].

The significant (*p* ≤ 0.05) increase in drug dissolution for Ivm-NC compared to Ivm coarse suspension starting over the 4–24 h period can be explained in light of the crystalline/amorphous character of both raw Ivm and Ivm-NC (Sect. "Powder X-ray diffraction (XRD)"). Indeed, XRD analysis confirmed the amorphous state of Ivm-NC, which likely contributed to enhanced Ivm dissolution compared to crystalline Ivm coarse suspension. In addition, the nanosize of Ivm-NC conferring a high surface area further boosted drug dissolution compared to unprocessed Ivm coarse suspension [63, 64].

### In vitro selective anticancer activity

In vitro anti breast cancer efficacy of the innovative, biomimetic camouflaged PMV/Ivm-NC was investigated on the aggressive triple-negative breast cancer cell line MDA-MB 231. Selective in vitro cytotoxicity, antimigration potential, cancer cell affinity and proapoptotic activity were elaborated.

#### Selective in vitro anticancer cytotoxicity

**Fig. 5:**
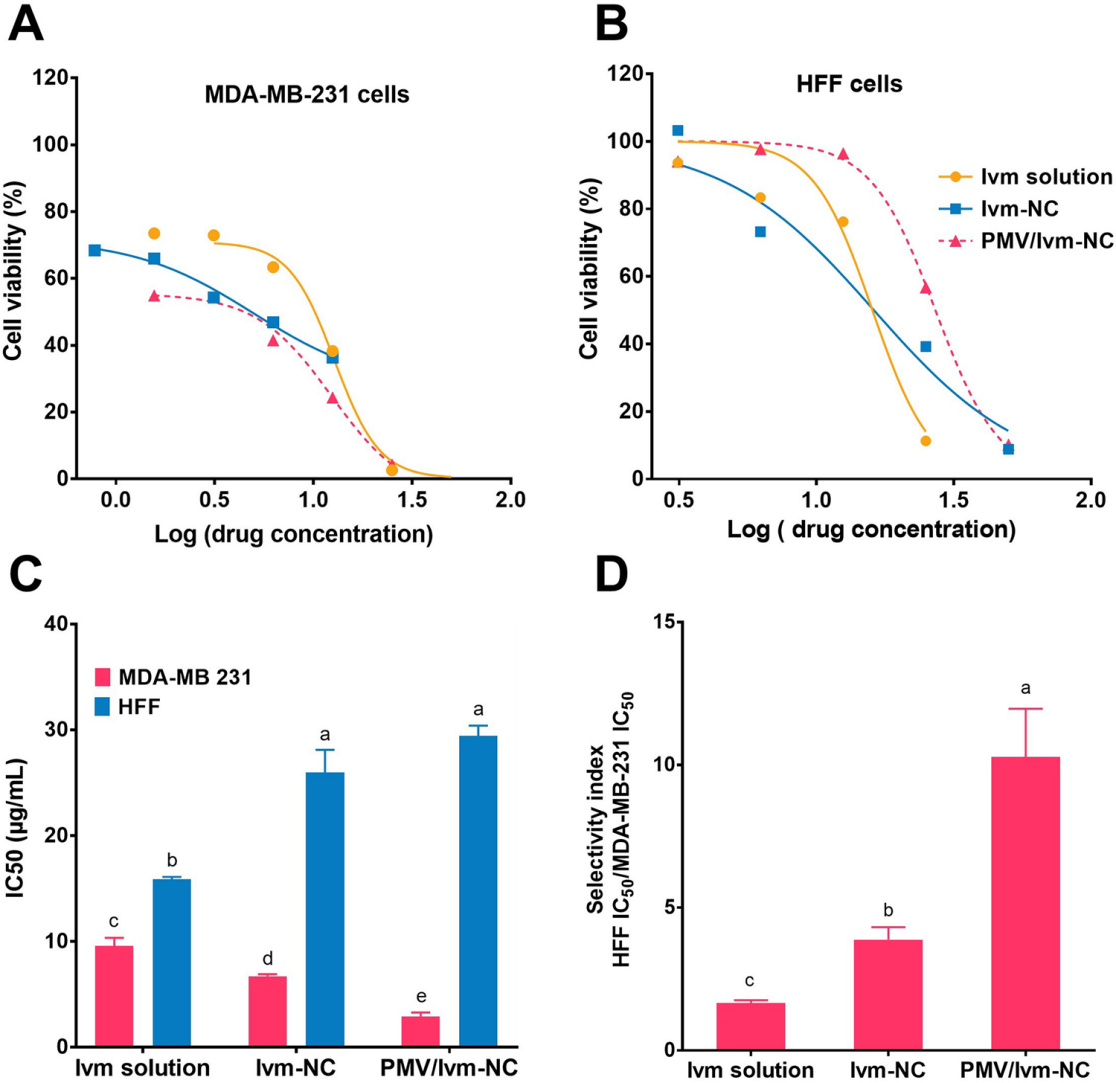
Selective in vitro cytotoxic activity of the developed nanocrystals on triple-negative breast cancer cells compared to normal cells (**A**–**D**). Viability curves of MDA-MB-231 (**A**) and HFF (**B**) after treatment with nanocrystals (at 0.78–25 µg/mL drug concentration) for 48 h. The calculated IC_50_ values for nanocrystals (**C**) and the cancer selectivity index (**D**) for MDA-MB-231 vs. HFF cells, verifying the pronounced selective cytotoxicity of PMV/Ivm-NC on cancer cells compared to normal cells. Data represents mean ± SD, n = 4. Letters indicate statistically significant difference at *p* ≤ 0.05: a > b > c > d > e

The antiproliferative effect of Ivm-NC and PMV/Ivm-NC compared to Ivm solution on MDA-MB-231 cells was evaluated using the MTT assay (Fig. 5A**)**. In addition, cytocompatibility was investigated on human foreskin fibroblasts (HFF; Fig. 5B). The IC_50_ of different formulations was recorded for both cell types (Fig. 5C**)**, and a selectivity index was calculated (Fig. 5D**)**.

Results showed that all the applied treatments exerted a dose-dependent antiproliferative effect on MDA-MB-231 cells over the tested concentration range (corresponding to 0.78–25 µg/mL Ivm; Fig. 5A**)**. Specifically, the IC_50_ values significantly (*p* ≤ 0.05) decreased in the order: Ivm solution (9.58 ± 0.75 µg/mL) > Ivm-NC (6.70 ± 0.20 µg/mL) > PMV/Ivm-NC (2.89 ± 0.38 µg/mL; Fig. 5C). Similar IC_50_ values for Ivm solution were reported in previous studies, recording an IC_50_ of 8.8 µM (~ 7.7 µg/ml) [65] and 6 µM (~ 5.25 µg/ml) [66] on MDA-MB-231 cells. The anticancer cytotoxicity of Ivm can be related to the drug ability to influence key regulatory pathways such apoptosis, cell cycle progression and autophagy [6, 10, 65, 67].

The augmented anticancer cytotoxicity of Ivm-NC compared to Ivm solution (1.4-fold decrease in IC_50_ value) can be primarily attributed to their endocytosis-driven uptake, which allows greater intracellular accumulation than the passive diffusion pathway typical for drug solutions [68]. Once internalized, NC undergo gradual dissolution, sustaining elevated intracellular drug levels and enhancing cytotoxic effects [69]. These findings align with previous reports showing that nanocrystal formulations often outperform their solubilized counterparts, due to the superior cellular interaction and uptake [70]. For instance, P188-stabilized niclosamide nanocrystals demonstrated enhanced cytotoxicity in MDA-MB-231, attributed to more efficient endocytic internalization [68]. Likewise, P188-coated paclitaxel nanocrystals demonstrated greater intracellular accumulation over Taxol® [69], confirming the combined role of nanocrystal structure and surface functionalization in enhancing therapeutic efficacy.

Further biomimetic PMV functionalization afforded a prominent increase in anticancer activity (with a 3.2-fold decrease in IC_50_ value compared to solution, and 2.3-fold compared to uncoated NC). This finding can be attributed to the enhanced intracellular uptake via receptor-mediated endocytosis secured by CD62P (P-selectin) platelet membrane protein interaction with CD44 receptors [18, 71], overexpressed on MDA-MB-231 cells [39].

The viability curves for HFF (Fig. 5B**)** illustrated high percentage viability for PMV/Ivm-NC. Indeed, the HFF IC_50_ significantly (*p* ≤ 0.05) increased as follows: Ivm solution (15.86 ± 0.23 µg/mL), Ivm-NC (25.97 ± 2.13 µg/mL) and PMV/Ivm-NC (29.43 ± 0.97 µg/mL; Fig. 5C**).** This pattern is further evidenced by the selectivity index values (Fig. 5D**)**, defined as the ratio of IC_50_ value recorded for normal cells to that for cancer cells. Results indicated the significantly (*p* ≤ 0.05) highest selectivity obtained for PVM/Ivm-NC (10.27 ± 1.68), reflecting a substantially improved therapeutic window. The prominent cytocompatibility endowed by PMV camouflage functionalization supports the preferential receptor-mediated uptake by cancer cells compared to normal cells, eventually contributing high normal cell cytocompatibility. It is worth mentioning that the relative Ivm intrinsic selectivity toward cancer cells might arise from differential expression of Ivm-sensitive pathways compared to normal cells. For instance, ivermectin has been shown to induce PAK1 degradation in breast cancer cells [10], mitochondrial dysfunction in renal cancer cells [72] and increased chloride intracellular influx in leukemia cells [73], targeting structures that are overexpressed in cancer cells, compared to normal cells. This selectivity was further enhanced in our study after nanocrystallization due to enhanced uptake by endocytosis, and further with PMV coating due to P-selectin-CD44 mediated interaction.

Our findings verify the augmented cancer cytotoxicity, anticancer selectivity and normal cell cytocompatibility endowed by Ivm repurposing, nanocrystallization and further biomimetic PMV functionalization, highlighting PMV/Ivm-NC as promising platform for anticancer functionality against triple negative breast cancer.

#### Cancer cell affinity and internalization

To evaluate the effect of nanocrystallization and PMV functionalization on cancer cellular uptake, coumarin-6 (C6) dye was employed as a model lipophilic fluorescent probe for the preparation of NC (C6-NC) and PMV/C6-NC followed by confocal microscopy visualization.

**Fig. 6:**
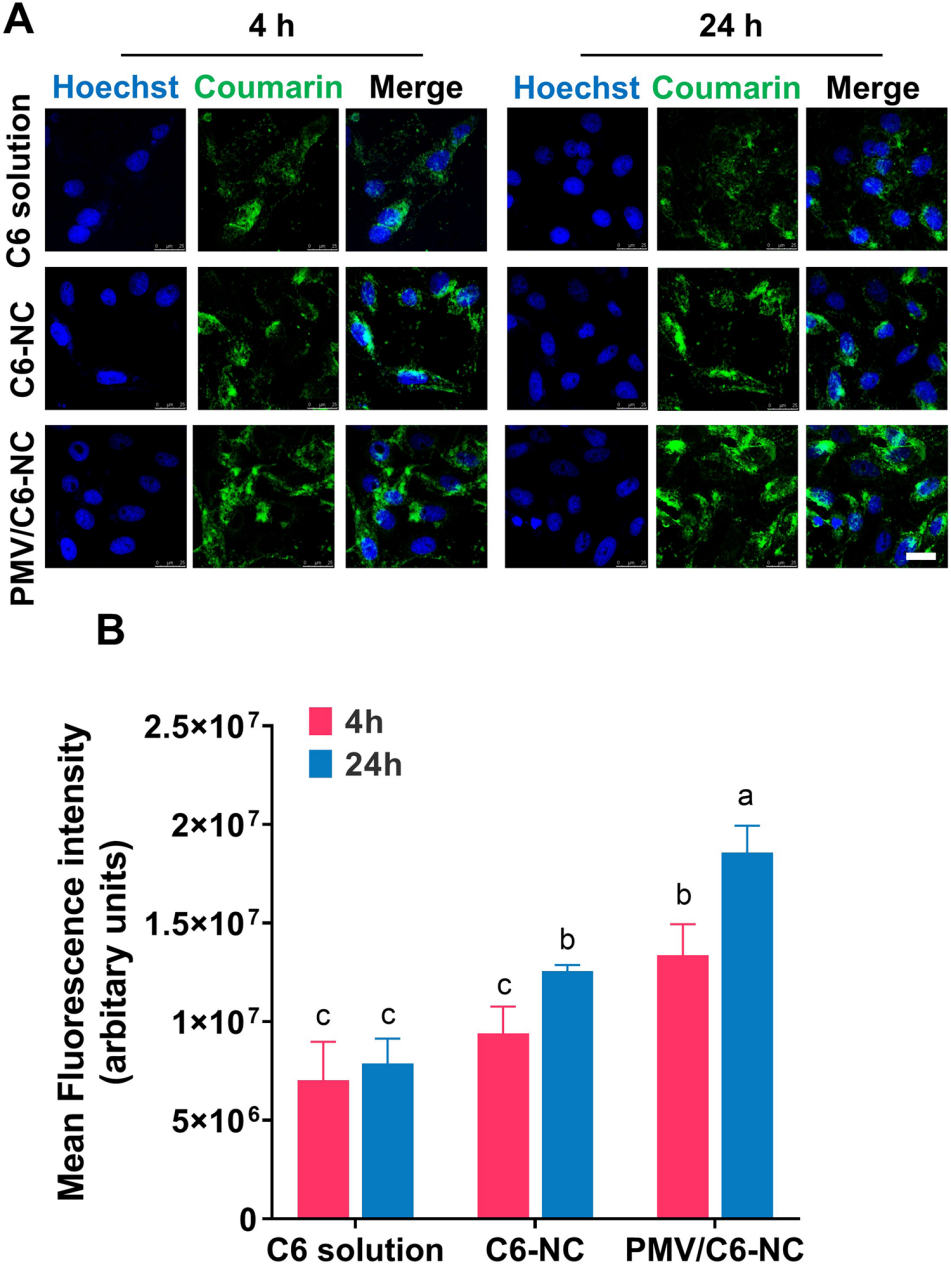
In vitro intracellular uptake of different nanocrystals by MDA-MB 231 after incubation for different time intervals (**A** and **B**). Representative CLSM images of cellular internalization of the developed C6 nanocrystals (green) against Hoechst (blue)-labeled nuclei (**A**), scale bar = 25 µm. Quantification of fluorescence intensity (**B**). Data represent mean ± SD, n = 4. Letters indicate statistically significant difference at *p* ≤ 0.05: a > b > c

As shown in Fig. 6Aand B, C6 solution demonstrated similar fluorescence intensities after both 4 and 24 h, indicating the absence of time-dependent intracellular uptake and the attainment of equilibrium condition [74, 75], a state which might be explained by C6 inherent uptake mechanism. Specifically, C6 is a lipophilic molecule that penetrates cells mainly via passive diffusion. This process greatly depends on the concentration gradient across the cell membrane, where diffusion stops when a concentration equilibrium is reached [39], and hence minimal time-dependent uptake was noted. In addition, the uptake of C6 may be limited by the presence of efflux pumps that can actively expel bare molecules from the cell [74]. Nanocrystallization was associated with an increase (*p* ≤ 0.05) in fluorescence intensity after 24 h, with a 1.3-fold increase compared to 4 h. Similar to a previous report [28], NC are hypothesized to undergo higher cell interaction and endocytosis-driven uptake compared to free solution.

A pronounced (*p* ≤ 0.05), time dependent increase in fluorescence intensity was afforded by PMV/C6-NC (Fig. 6B**)**, with a 1.5-fold increase compared to C6-NC after 24 h. This finding can be attributed to receptor-mediated endocytosis, facilitated by the interaction between the P-selectin platelet membrane protein and cancer cell CD44 receptors, promoting internalization and accumulation of the fluorescent cargo within the cell [76]. This finding corroborates and justifies the selective cytotoxicity exerted by biomimetic PMV functionalization (Sect. "Selective in vitro anticancer cytotoxicity").

#### Cell migration inhibition

The antimigration activity of Ivm-NC and PMV/Ivm-NC compared to Ivm solution was tested on MDA-MB-231 cells.

**Fig. 7:**
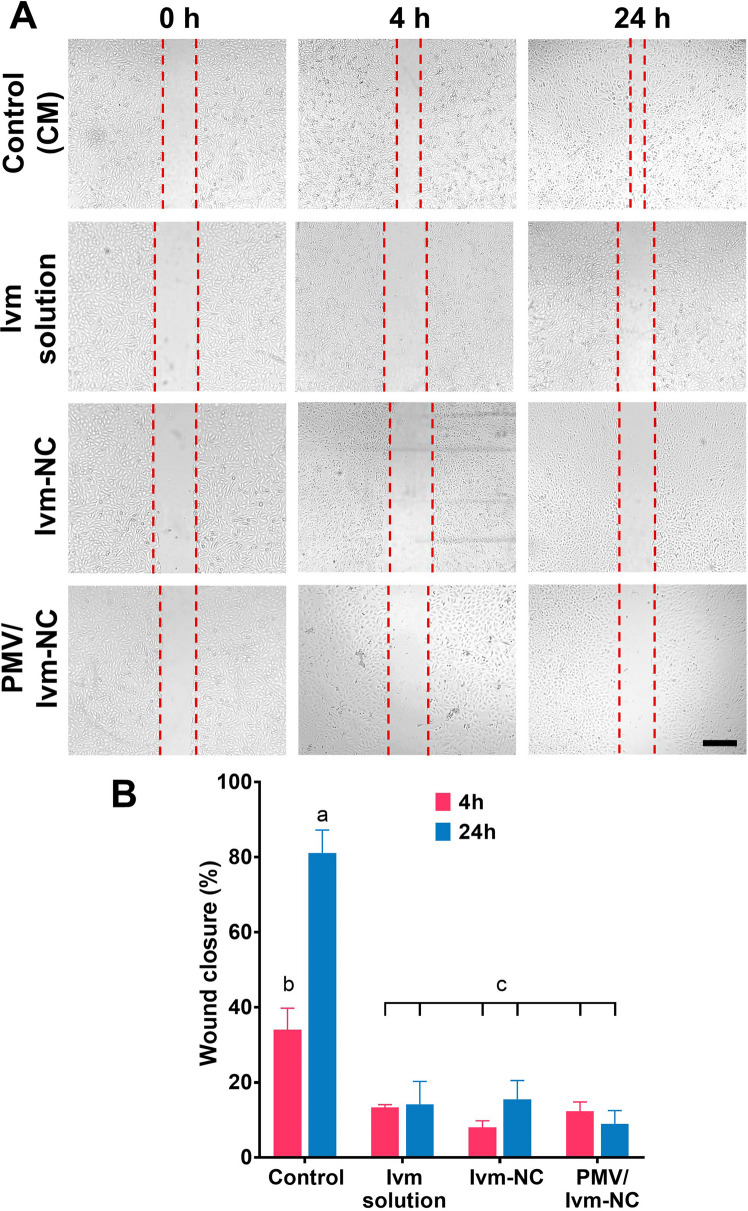
In vitro antimigratory activity of the developed nanocrystals (at half their IC_50_) on MDA-MB 231 after treatment for different time intervals (**A** and **B**). Representative microscope images of the scratch borders (dotted red lines) in the cell monolayer (**A**), scale bar = 200 µm. Percentage wound closure (**B**). Data represents mean ± SD, n = 4. Letters indicate statistically significant difference at *p* ≤ 0.05: a > b > c

Results demonstrated that all treatment samples exerted a significantly (*p* ≤ 0.05) higher antimigration activity compared to control at both 4 h and 24 h (Fig. 7A and B). Indeed, control cells exhibited 81.16 ± 6.09% wound closure at 24 h, while treatment samples afforded an average of 12.88% closure. No significant differences were observed among the treated groups at either time point, suggesting that the pronounced anti-migration effect of Ivm was completely realized regardless of formulation. These findings align with previous studies reporting that Ivm inhibits the migration of triple-negative breast cancer cells [77] via regulation of the expression of migration-associated proteins such as inhibition of matrix metalloproteinase 9 (MMP 9), and upregulation of epithelial cells markers such as E-cadherin. In addition, Ivm has been reported to inhibit Wnt/β-catenin and integrin β1/FAK signalling pathways, which are involved in the expression of metastasis-related proteins, in multiple cell lines including MCF-7 breast cancer and HCT colon cancer carcinoma cells [77].

#### Apoptosis activity

The proapoptotic activity of Ivm-NC and PMV/Ivm-NC on MDA-MB-231 cells was elaborated via the Annexin V-FITC/PI assay.

**Fig. 8:**
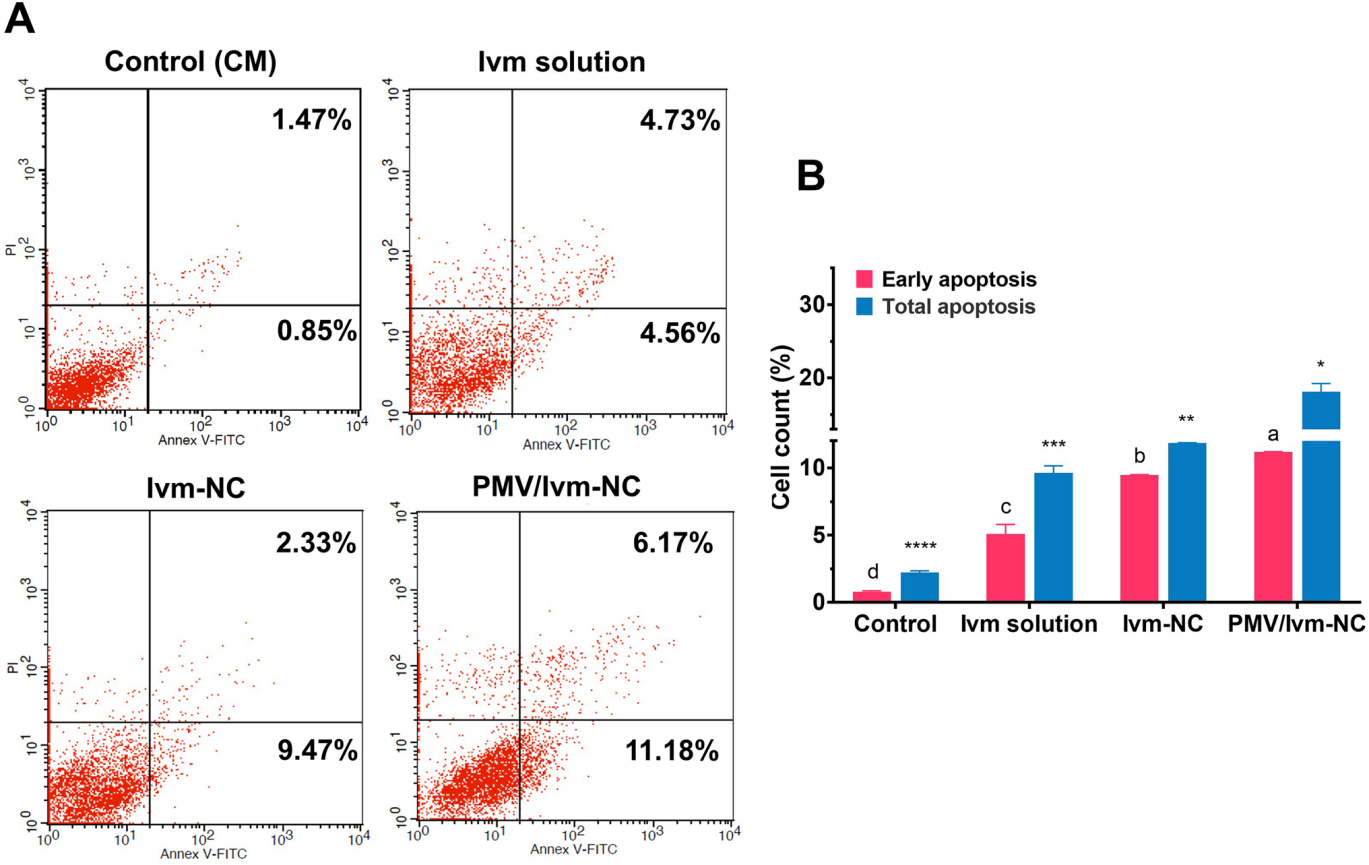
In vitro proapoptotic activity of the developed nanocrystals (at half their IC_50_) on MDA-MB 231 (**A** and **B**). Representative flow cytometry scatter plots for Annexin-V FITC/propidium iodide (**A**) and quantification of cell count (**B**), verifying the prominent early proapoptotic activity of PMV/Ivm-NC. Data represents mean ± SD, n = 3. Letters and symbols indicate statistically significant difference at *p* ≤ 0.05: a > b > c > d and * > ** > *** > ****

As illustrated in Fig. 8A, all treatment samples featured a higher population of cells undergoing early apoptosis compared to control cells, with a parallel increase in total apoptosis. In particular, the percentage early apoptosis increased in the order: Ivm solution (5.07 ± 0.52%) < Ivm-NC (9.17 ± 0.29%) < PMV/Ivm-NC (11.02 ± 0.16%; Fig. 8B**).** The proapoptotic potential of Ivm obtained in this work is consistent with a previous study by Güler et al. [78], which reported a dose-dependent proapoptotic effect of Ivm on MDA-MB-231 cells. It has been established that Ivm exerts a pro-apoptotic effect by generating reactive oxygen species, causing mitochondrial membrane depolarization and ATP depletion, ultimately resulting in apoptotic cell death [78, 79].

The nanocrystallization-associated enhanced proapoptotic effect for Ivm-NC is possibly referred to improved cell interaction and controlled release. Furthermore, biomimetic PMV functionalization contributed substantial enhancement of proapoptotic activity for PMV/Ivm-NC, probably due to the higher cell interaction and receptor-mediated uptake and internalization. These results collectively corroborate cytotoxicity findings **(**Sect. "Selective in vitro anticancer cytotoxicity").

Taken together, our results underscore biomimetic PMV/Ivm-NC for the prominently selective cancer cytotoxicity, enhanced cancer cell internalization and proapoptotic potential with high normal cell cytocompatibility.

### Hemocompatibility studies

For the verification of PMV/Ivm-NC hemocompatibility and clinical biosafety, in vitro hemolysis and in vivo tail bleeding testing were performed.

The in vitro hemolysis assay was conducted to assess the hemocompatibility of Ivm-NC and PV/Ivm-NC, intended to be given by intravenous administration. Positive and negative control samples (100% and 0% hemolysis, respectively) were assigned. Visual inspection and spectrophotometric quantification of hemolyzed hemoglobin were performed.

Results demonstrated a hemolytic effect of Ivm-NC (Figure S2(A), as inferred by percentage hemolysis value (57.97 ± 0.68%**; **Figure S2(B)). This result is consistent with a previous report establishing RBC membrane disruption by Ivm [80]. Favorably, PMV camouflage functionalization notably ameliorated Ivm-inherent hemolytic activity, affording the least (*p* ≤ 0.05) percentage hemolysis (6.47 ± 2.59%; Figure S2(B)). This finding clearly highlights the improved hemocompatibility endowed by PMV functionalization, probably attributed to the biomimetic shielding effect of PMV on the surface of Ivm-NC. Such bioderived camouflage mimics native platelet surface, minimizing offensive interaction with erythrocyte membranes and thereby improving hemocompatibility. Similarly, Gao et al. [81] reported lower percentage hemolysis upon coating genistein-loaded liposomes with platelet membrane compared to uncoated vesicles.

The in vivo tail bleeding assay was performed to evaluate the effect of nanocrystallization and PVM camouflage functionalization on in vivo hemostasis in BALB/c mice. To this end, the time required for animal severed tails to stop bleeding was reported for all groups, including Ivm-NC and PMV/Ivm-NC, compared to untreated animals (control). There was no difference (*p* > 0.05) between the recorded bleeding time across all treatment groups, with the range 1.5–2.0 min (**Figure S2(C)**), indicating no substantial effect of different samples on in vivo hemostasis.

Taken together, our findings verify the hemocompatibility of the innovative, biomimetic camouflaged PMV/Ivm-NC, showing almost no interference with in vivo hemostasis (either pro- or anti-coagulant effects). Such results highlight opportune PMV functionalization for augmented anticancer activity with high in vivo compatibility.

### In vivo tumor targeting and tissue distribution

The impact of biomimetic PMV functionalization on in vivo distribution and tumor homing was elaborated in an orthotopic 4T1 breast cancer model, employing C6 for the preparation of C6-NC and PMV/C6-NC. To this end, fluorescence was recorded and quantified in tumor tissue as well as major organs.

**Fig. 9:**
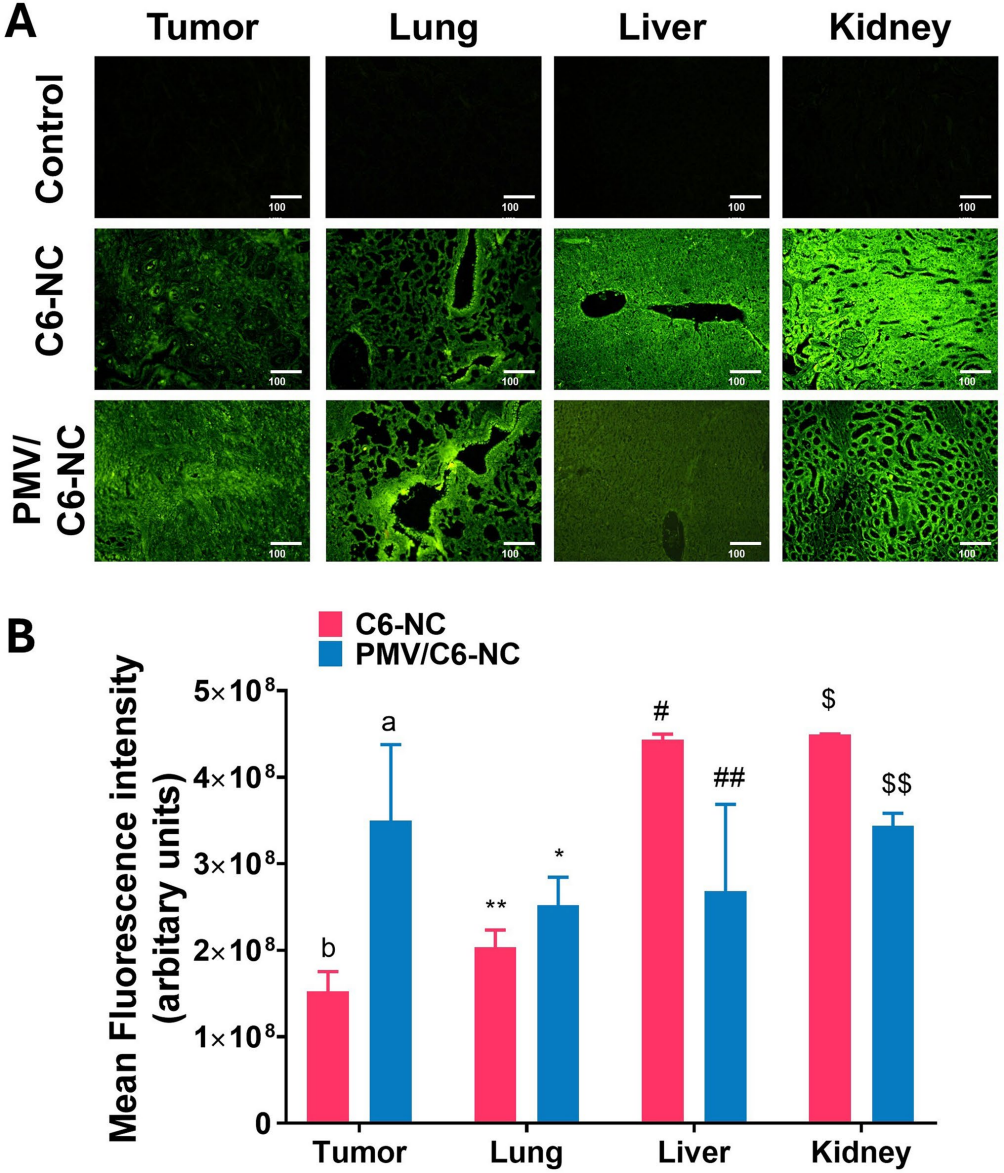
Biodistribution of coumarin-labeled nanocrystals (C6-NC) and platelet membrane vesicles-coated C6- nanocrystals (PMV/C6-NCs) in a 4T1 breast cancer mouse model. Confocal laser scanning microscopy (CLSM) images of tumor, lung, liver, and kidney tissues showing fluorescence distribution of C6-NC and PMV/C6-NC (**A**). Quantification of mean fluorescence intensity in each organ (**B**). Data represents mean ± SD (*n* = 4). Letters and symbols indicate statistically significant differences at *p* ≤ 0.05. a > b; * > **; # > ##; $ > $$

The biodistribution analysis revealed a marked difference in organ-specific accumulation (Fig. 9A and B). The biomimetic camouflaged PMV/C6-NC afforded a significant (*p* ≤ 0.05) 2.3-fold increase in fluorescence intensity of tumor tissues compared to uncoated C6-NC, indicating enhanced active targeted tumor homing. In parallel, the liver and kidney accumulation of PMV/C6-NC presented a 1.65-fold and 1.3-fold reduction, respectively, compared to uncoated C6-NC, highlighting a favorable reduction of off-target tissue distribution and hence improved systemic safety. These findings are consistent with previous reports where platelet membrane coating of PLGA nanoparticles resulted in enhanced breast cancer accumulation with minimal liver and kidney distribution, compared to uncoated nanoparticles [82, 83]. Notably, previous reports on platelet membrane-coated nanocarriers consistently showed enhanced accumulation at disease sites, without preferential uptake in other healthy organs such as heart or spleen, supporting the absence of off-target accumulation upon platelet membrane coating [84, 85].

This selective tumor homing can be attributed to the biomimetic properties conferred by the platelet membrane coating, which potentially facilitates active targeting via CD44-P-selectin receptor-ligand interactions and enhances circulation time by evading phagocytic clearance [86]. The reduced liver and kidney uptake likely reflects diminished recognition by the mononuclear phagocyte system, decreasing nonspecific organ distribution and potential off-target toxicity. This immune evasion could be attributed to the CD47 expression on the platelet membrane, which interacts with SIRPα receptors on macrophages to deliver inhibitory `don’t eat me´ signals that suppress phagocytosis, in addition to a potential reduction in protein adsorption in blood, thereby reducing opsonization and subsequent recognition by reticuloendothelial system [87].

In conclusion, our findings establish the high tumor homing endowed by biomimetic-camouflage, PMV functionalization for pronounced tumor targeting, minimal off-target distribution and prominent antitumor functionality.

### In vivo antitumor efficacy in an orthotopic 4T1 triple negative breast cancer

The antitumor potential and safety profile of Ivm solution, Ivm-NC and biomimetic PMV/Ivm-NC were evaluated through a comprehensive in vivo study in an orthotopic 4T1 breast cancer murine model. Anticancer activity was assessed through tumor growth monitoring and histopathological and morphometric analysis. Mechanistic insights were elaborated for tumor apoptosis, angiogenesis and cell cycle regulation via ELISA and RT-qPCR. Moreover, immunohistochemical analysis of immunotherapeutic anticancer activity was investigated. Safety evaluation included body weight monitoring, blood analysis and major organ histopathology. The lung antimetastatic potential was also elaborated.

#### Tumor growth inhibition

**Fig. 10:**
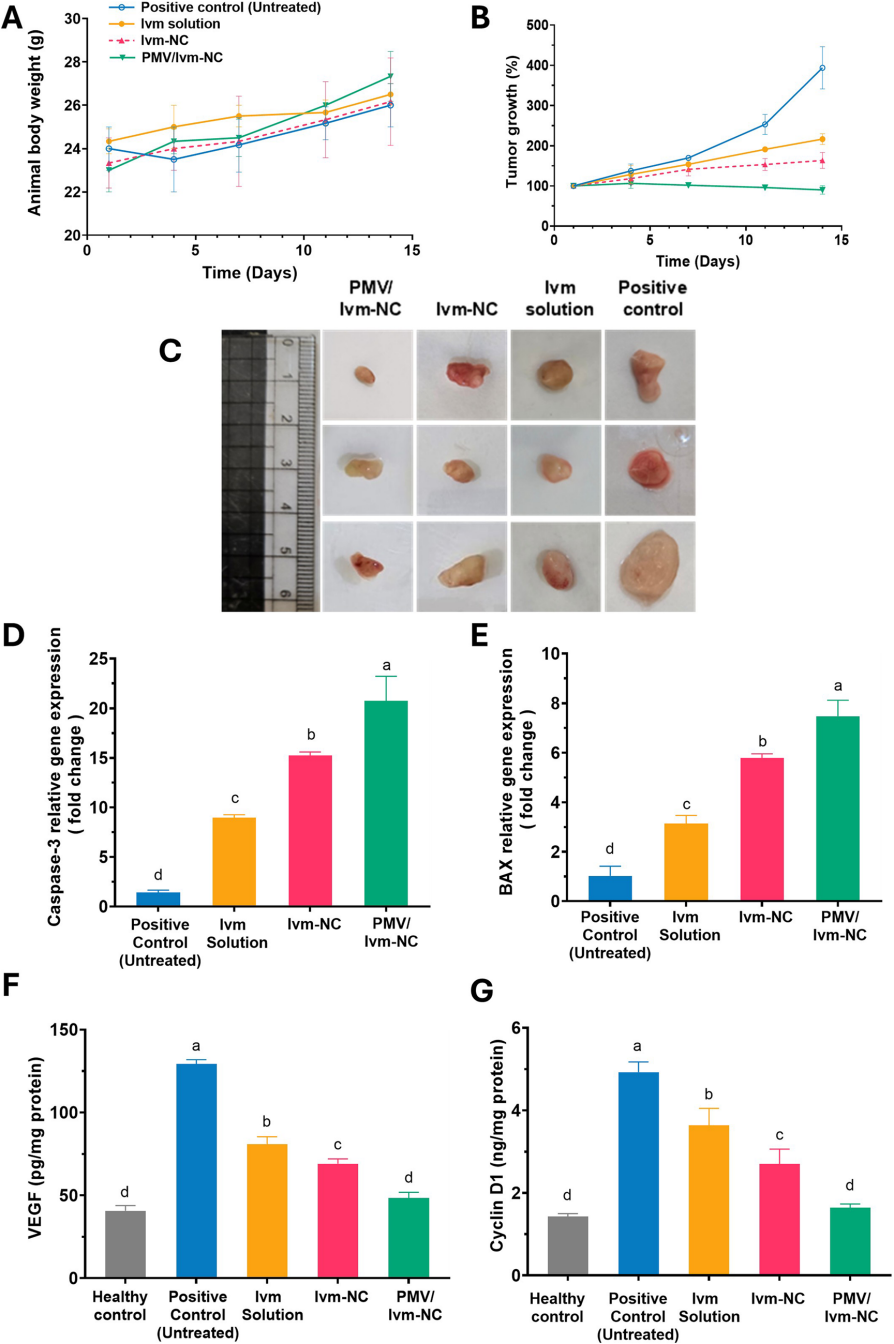
In vivo antitumor therapeutic efficacy and mechanistic analysis of Ivm NC formulations in 4T1 breast cancer-bearing BALB/c mice (**A**-**G**). Body weight monitoring (**A**) and tumor volume changes (**B**) expressed as percentage change from baseline of mice throughout the treatment period. Representative photographs of excised tumors from each treatment group at the study endpoint(**C**). RT-qPCR analysis of mRNA expression levels of pro-apoptotic genes Caspase-3 (**C**) and Bax (**D**). ELISA quantification of VEGF (**E)** and cyclin D1 (**F**) protein levels. Data are presented as mean ± SD (n = 3). Letters and symbols indicate a statistically significant difference at *p* ≤ 0.05. a > b > c > d

Throughout the treatment period, all animal groups exhibited normal and steady body weight gain with no significant (*p* > 0.05) differences observed between treated and control groups (Fig. 10A), suggesting that the administered formulations did not adversely impact animal growth or general health.

As shown in Fig. 10B and C, treatment with Ivm formulations significantly (*p* ≤ 0.05) inhibited tumor growth, compared to the control group. Specifically, the untreated group (receiving saline), showed a steady increase in tumor volume throughout the experiment, striking a 393.8 ± 52.5% tumor growth by day 14-post treatment. In contrast, Ivm group demonstrated a favorable decline (*p* ≤ 0.05) in tumor growth (216.4 ± 13.2%) by day 14-post treatment, confirming the antitumor efficacy of Ivm against triple negative breast cancer model as previously reported [10]. The 1.3-fold decrease of tumor volume with Ivm-NC compared to Ivm solution might suggest an improved accumulation in tumor tissue, which may be attributed to endocytosis-mediated cellular uptake of NC compared to passive diffusion of free drug solution [68]*.* In addition, the nanosize-mediated EPR effect might have contributed an improved tumor localization. Biomimetic PMV functionalization afforded a 1.8-fold reduction in the 14-day tumor volume compared to uncoated Ivm-NC. This pattern can be possibly attributed to the active targeted tumor homing and prolonged circulation secured by PMV coating and consequent P-selectin/CD44 interaction. Similar findings were reported by Song et al. where platelet membrane coated paclitaxel-PLGA nanoparticles showed higher antitumor efficacy against breast cancer, compared to paclitaxel solution and uncoated nanoparticles. This effect may be attributed to the tumor targeting properties of platelet membrane coating [83]. These results are supported by the tumor homing study results (Sect. "In vivo tumor targeting and tissue distribution") which showed enhanced tumor accumulation on PMV functionalization.

Collectively, our findings highlight the superior antitumor efficacy of the platelet-mimetic, camouflaged PMV/Ivm-NC for effective tumor growth inhibition in triple negative breast cancer.

#### Tumor apoptosis, angiogenesis and cell cycle regulation

Apoptosis, or programmed cell death, is a tightly regulated cellular process that serves as a natural barrier against cancer development. However, in cancer, including breast cancer, this mechanism is often suppressed, enabling uncontrolled cell proliferation. Key regulators of the intrinsic apoptotic pathway include caspase-3 which is a critical executioner of apoptosis that is responsible for the cleavage of vital cellular components during apoptosis, and Bax, a pro-apoptotic protein that promotes mitochondrial membrane permeabilization [88]. To evaluate the possible pro-apoptotic effects of the tested NC formulations, the mRNA expression of caspase-3 and Bax were quantified in excised tumor tissues using RT-qPCR.

As shown in Fig. 10D and E, both markers were significantly upregulated in the treated groups compared to untreated tumor-bearing groups (*p* ≤ 0.05). In particular, both caspase-3 and Bax upregulation (*p* ≤ 0.05) followed the pattern: PMV/Ivm-NC > Ivm-NC > Ivm solution. These results indicate the antitumor efficacy of Ivm treatment via Bax-associated enhanced mitochondrial-mediated apoptosis as well as caspase-3-related activation of downstream apoptotic signaling pathways. These findings correlate with the in vitro apoptosis assay for the tested treatments (Sect. "Apoptosis activity"), which confirms the pro-apoptotic effect of Ivm. Our results are in agreement with previous reports demonstrating that Ivm induces apoptosis in cancer cells. For instance, Draganov et al. reported Ivm-induced activation of caspase-3 in 4T1 breast cancer cells, where cell-death was inhibited upon using caspase inhibitors, confirming the pro-apoptotic mechanism of Ivm [89]. In addition, Ivm has shown upregulation of apoptosis markers, including Bax and caspase-3, in a number of cell lines including cervical cancer cell line (Hela) [90] and leukemia cell lines (K562) [41].

The two key tumor biomarkers (VEGF and cyclin D1) were further quantified via ELISA. Vascular endothelial growth factor (VEGF) is a key pro-angiogenic molecule that is upregulated in tumor tissues compared to healthy counterparts [91], promoting the formation of abnormal leaky vasculature that facilitates tumor growth, invasion and metastasis [92]. On the other hand, cyclin D1 is a critical cell cycle regulator whose expression is tightly controlled in normal cells, but frequently overexpressed in breast cancer, where it drives uncontrolled proliferation and serves as a marker of poor prognosis [93].

As illustrated in Fig. 10F and G, there was a significant (*p* ≤ 0.05) knockdown of both VEGF and cyclin D1 for all treatment groups, compared to the untreated control group. The most prominent downregulation (*p* ≤ 0.05) was afforded by the biomimetic PMV/Ivm-NC group for VEGF (48.33 ± 3.51 pg/mg) and cyclin D1 (1.64 ± 0.09 ng/mg). The marked decrease in VEGF levels observed highlights the antiangiogenic potential of Ivm-based treatments. These results are in accordance with a previous in vivo study in BALB/c mice with non-small cell lung cancer, where Ivm significantly reduced pulmonary VEGF levels following oral Ivm administration [94]. Our results further align with prior studies demonstrating IV ability to reduce cyclin D1 expression, alongside other cell cycle regulators such as cyclin E and PCNA [66], in various breast cancer models [66], colorectal (HT29) and lung (H358) cancer cells [95], suggesting cell cycle arrest at the G0/G1-S checkpoint.

The nanocrystallization-conferred potentiation of cancer proapoptotic and antiangiogenic activities and cell cycle regulation are possibly the result of combined nanocarrier-associated enhanced cell interaction, nanoparticle-mediated endocytosis and EPR effect. In addition, the superiority of antitumor activity endowed by PMV biomimetic camouflage can be further assigned to improved tumor targeting/homing and cellular uptake (Sect. "In vivo tumor targeting and tissue distribution") via interaction between the P-selectin platelet membrane protein and cancer cell CD44 receptors, overexpressed in cancer cells.

#### Histopathological and histomorphometric analysis

**Fig. 11:**
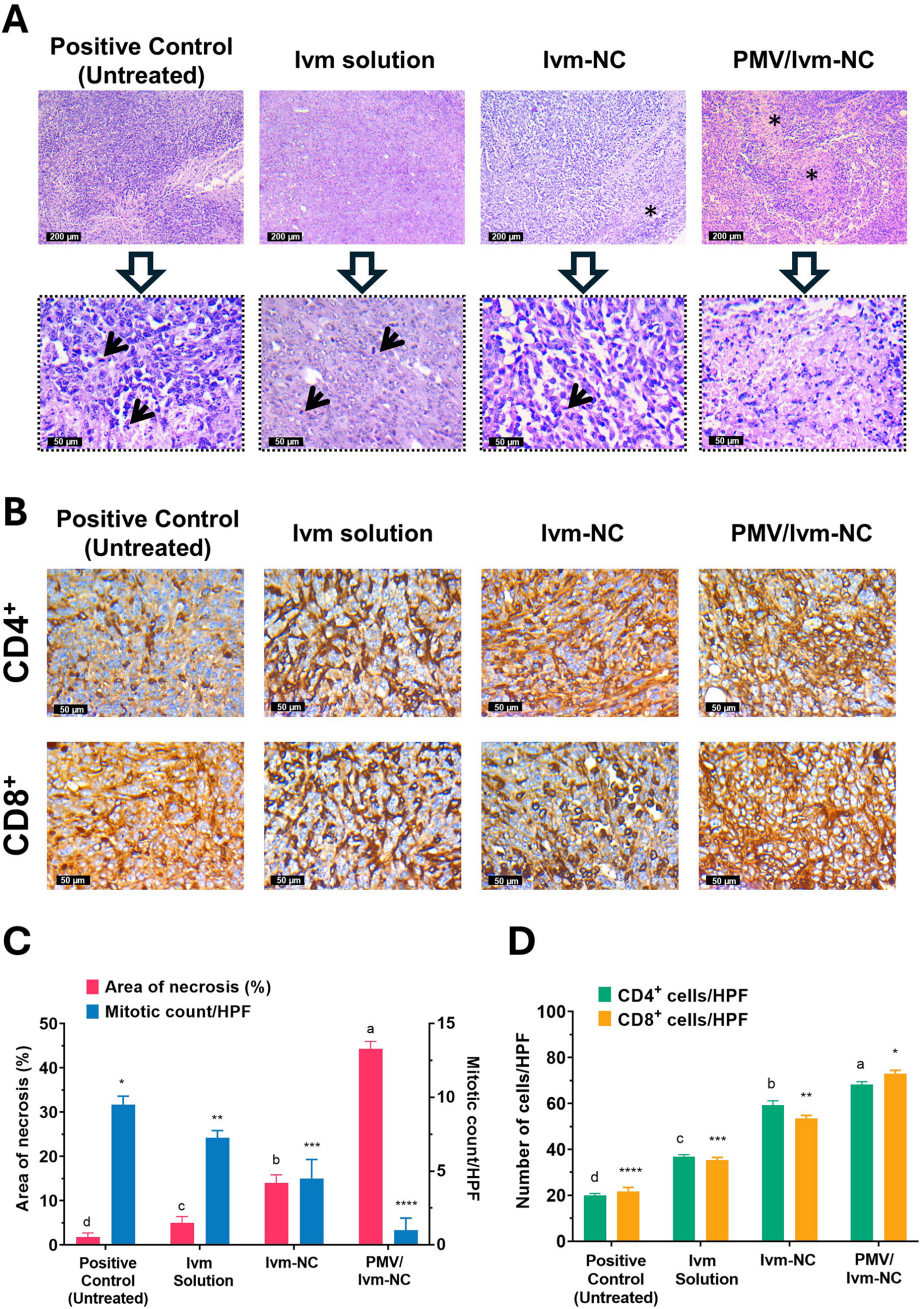
Histological, morphometric and immunohistochemical analysis of breast tumor tissue in different groups following treatment (**A**–**D**). Representative H&E-stained images for different groups at low power (× 100) and high power (× 400)**; A)**. Positive control (untreated group) shows sheets of cohesive, spindle to polygonal tumor cells with minimal necrosis. High power shows highly pleomorphic cells with frequent mitotic figures (arrows). Similar findings were seen in Ivm solution group with mitotic figures still detected on high power (arrows). Meanwhile, areas of necrosis (*) are seen in Ivm-NC and PMV/Ivm-NC groups. High power shows apoptotic cells and cellular debris with occasional mitotic figures (arrow). Representative images of immune-stained sections for assessment of tumor-infiltrating CD4^+^ and CD8^+^ T lymphocytes **(B)**, where positive cells are seen as brown cytoplasmic stained cells within tumor cell population. The count was minimal in positive control and slightly increased in Ivm solution. The count of positive-stained cells was much higher in Ivm-NC and PMV/Ivm-NC treated groups. (IHC, × 400). Morphometric analysis of H&E-stained sections for the quantification of necrosis areas and mitotic count **(C)**. Quantification of immune-positive cell count **(D)**. Data are presented as mean ± SD (n = 3). Letters and symbols indicate a statistically significant difference at *p* ≤ 0.05. a > b > c > d and * > ** > *** > ****

Histological investigations were performed for different animal groups to scrutinize the cytopathological features of tumor inhibition (Fig. 11A**)** and immunohistochemical assessment of immune markers (Fig. 11B**).** Quantification of tumor necrosis area and mitotic count in H&E-stained sections was also performed via image analysis (Fig. 11C**),** and immune markers were quantified (Fig. 11D**)**.

For animals of the positive control (untreated) group, sheets and nodules of cohesive tumor cells were seen infiltrating nearby fat and skeletal muscle fibers. Tumor cells were spindle to polygonal in shape showing vesicular nuclei with prominent nucleoli, while the cytoplasm was pale eosinophilic (Fig. 11A**)**. Numerous mitotic figures were frequently detected (9.5 ± 0.57), with very limited necrosis within the tumor (1.75 ± 0.95%; Fig. 11C**)**. Meanwhile, Ivm solution treated group showed a few inflammatory cells infiltration and scanty apoptotic bodies (high power; Fig. 11A**).** Several mitotic figures were still seen (7.25 ± 0.5) and minimal necrosis was detected (5 ± 1.41%). Animals of the Ivm-NC treated groups showed tumor cells with evident cytoplasmic prominent eosinophilia with pyknotic nuclei on the high power. There was an obvious decrease in mitotic cell count and increase of necrotic areas with numerous apoptotic bodies. Interestingly, the biomimetic PMV/Ivm-NC presented wide areas of necrosis, afforded a significant (*p* ≤ 0.05) 25.2-fold increase in tumor necrosis and a 9.5-fold decline in mitotic figure count compared to untreated animals (Fig. 11C**).** High power showed several apoptotic bodies.

Collectively, histological findings corroborate the promising antitumor activity and tumor growth inhibition (Sect. "Tumor growth inhibition") endowed by Ivm nanocrystallization and subsequent biomimetic PMV functionalization for Ivm-NC and PMV/Ivm-NC.

#### Antitumor immunotherapeutic activity

The tumor microenvironment of solid tumors is typically immunosuppressive, dominated by regulatory cells that enable immune evasion, with relatively low infiltration of effector T cells. Strategies that convert "cold" tumors (poor T cell infiltration) into "hot" tumors (rich in CD4^+^ helper and CD8^+^ cytotoxic T cells) are associated with enhanced responsiveness to immunotherapy [96]. Ivermectin has been reported to promote immunogenic cell death and stimulate antitumor immunity, leading to increased infiltration of CD4^+^ and CD8^+^ T cells, making their evaluation in tumors and important indicator of its immunotherapeutic activity [89, 97]. In our work, tumor infiltrating CD4^+^ and CD8^+^ T lymphocytes were quantified in different animal groups, where CD4^+^ and CD8^+^ cells were seen in the form of brown cytoplasmic staining (Fig. 11B and D**).** Positive cells were assessed within viable areas to avoid nonspecific staining in necrotic areas.

Results indicated a significant (*p* ≤ 0.05) increase in CD4^+^ and CD8^+^ T lymphocyte infiltration as follows: untreated group < Ivm solution < Ivm-NC < PMV/Ivm-NC. Indeed, biomimetic PVM functionalization achieved a ~ 3.4-fold increase in the counts of recruited CD4^+^ and CD8^+^ T lymphocyte compared to untreated mice. Similar results were previously reported for Ivm in vivo in a 4T1 mouse model of triple- negative breast cancer, where treatment was associated with increased infiltration of CD4^+^ and CD8^+^ T cells compared to control [97].

Our results collectively highlight the opportune immunotherapeutic antitumor repurposing of Ivm as promising candidate for triple negative breast cancer therapy. In addition, harnessing the biomimetic PMV functionalization positively contributed to the combined antitumor activity. It is worth mentioning that the augmented tumor suppression effect observed in vivo, compared to the modest apoptosis demonstrated in vitro can be attributed to the use of sub-cytotoxic concentrations and short exposure time in cell culture, which were selected to compare formulation mechanistically, rather than to maximize cell death. In vivo, repeated dosing every other day for 14 days provides higher and sustained exposure, which is expected to produce a stronger, time- and dose-dependent apoptotic response. Moreover, the in vivo study showed increased Bax and caspase-3, reduced VEGF and cyclin D1, and enhanced CD4^+^ and CD8^+^ T-cell infiltration, indicating that PMV/Ivm-NC exerts combined pro-apoptotic, antiangiogenic, antiproliferative and immunomodulatory effects that together explain the pronounced tumor suppression observed.

#### In vivo safety study

For the biocompatibility study, major organs (liver, kidney and spleen) were screened for histological alterations (**Figure S3**).

Results indicated that liver tissues of all animal groups showed preserved architecture. However, obvious lobular inflammation was seen in Ivm solution-treated group, a feature that was not detected in Ivm-NC and PMV/Ivm-NC treated groups (showing histologically free liver sections; **Figure S3)**. This finding indicates the favorable impact of nanocrystallization on boosting Ivm antitumor activity while mitigating possible hepatic adverse effects. These results are consistent with previous reports, where Ivm solution showed higher hepatotoxicity, compared to control and ivermectin-methyl dihydrojasmonate nanostructured lipid carrier [41].

Renal tissues showed preserved architecture of renal cortex, where glomeruli were histologically normal and seen against closely packed proximal tubules. No interstitial inflammation or fibrosis was detected. In addition, splenic tissues were histologically free in all treated groups. Lymphoid follicles of white pulp were seen within red pulp. No congestion or lymphoid hyperplasia were detected.

#### Serum biochemical markers for hepatic and renal function

To further evaluate the systemic safety of the tested formulations, serum biochemical markers of hepatic and renal functions were assessed, including alanine aminotransferase (ALT), aspartate aminotransferase (AST), alkaline phosphatase (ALP), urea and creatinine.

Across all treatment groups, no statistically significant differences (*p* ≥ 0.05) were observed, compared with the control saline group (Table 1), suggesting neither Ivm solution, nor Ivm-NC, with or without PMV coating, induced hepatic or renal toxicities. Collectively, these results indicate that the developed NC formulations are well tolerated in vivo with high biocompatibility and minimal systemic toxicity.

**Table 1:** Serum biochemical parameters for liver (ALT, AST, ALP) and kidney (urea, creatinine) function in mice following different treatments

Group	ALT (U/L)	AST (U/L)	ALP (U/L)	Urea (mg/dL)	Creatinine (mg/dL)
Control (untreated)	171.7 ± 41.0	570.6 ± 18.1	88.0 ± 12.7	63.6 ± 26.0	0.17 ± 0.02
Ivm solution	150.7 ± 29.5	227.3 ± 15.0	99.5 ± 41.8	49.7 ± 4.0	0.28 ± 0.12
Ivm-NC	135.3 ± 14.2	396.3 ± 91.7	83.0 ± 2.8	55.0 ± 7.8	0.27 ± 0.06
PMV/Ivm-NC	129.3 ± 7.6	492.0 ± 23.6	105.3 ± 20.1	51.7 ± 9.3	0.24 ± 0.02

Values are presented as mean ± SD (*n* = 5). No statistically significant differences were observed among groups (*p* > 0.05)

### In vivo antimetastatic potential

The 4T1 murine breast cancer model is a well-established model of triple-negative breast cancer, characterized by its aggressive growth and high propensity to metastasize, particularly to the lungs, thereby closely mimicking the clinical course of human triple negative breast cancer progression [98, 99]. To assess the antimetastatic potential of the different treatments, an intravenous 4T1 cell injection model was employed, which directly models the circulating tumor cell (CTC) phase of the metastatic cascade, providing an ideal platform for evaluating CTC-targeting and antimetastatic therapeutic strategies [44].

**Fig. 12:**
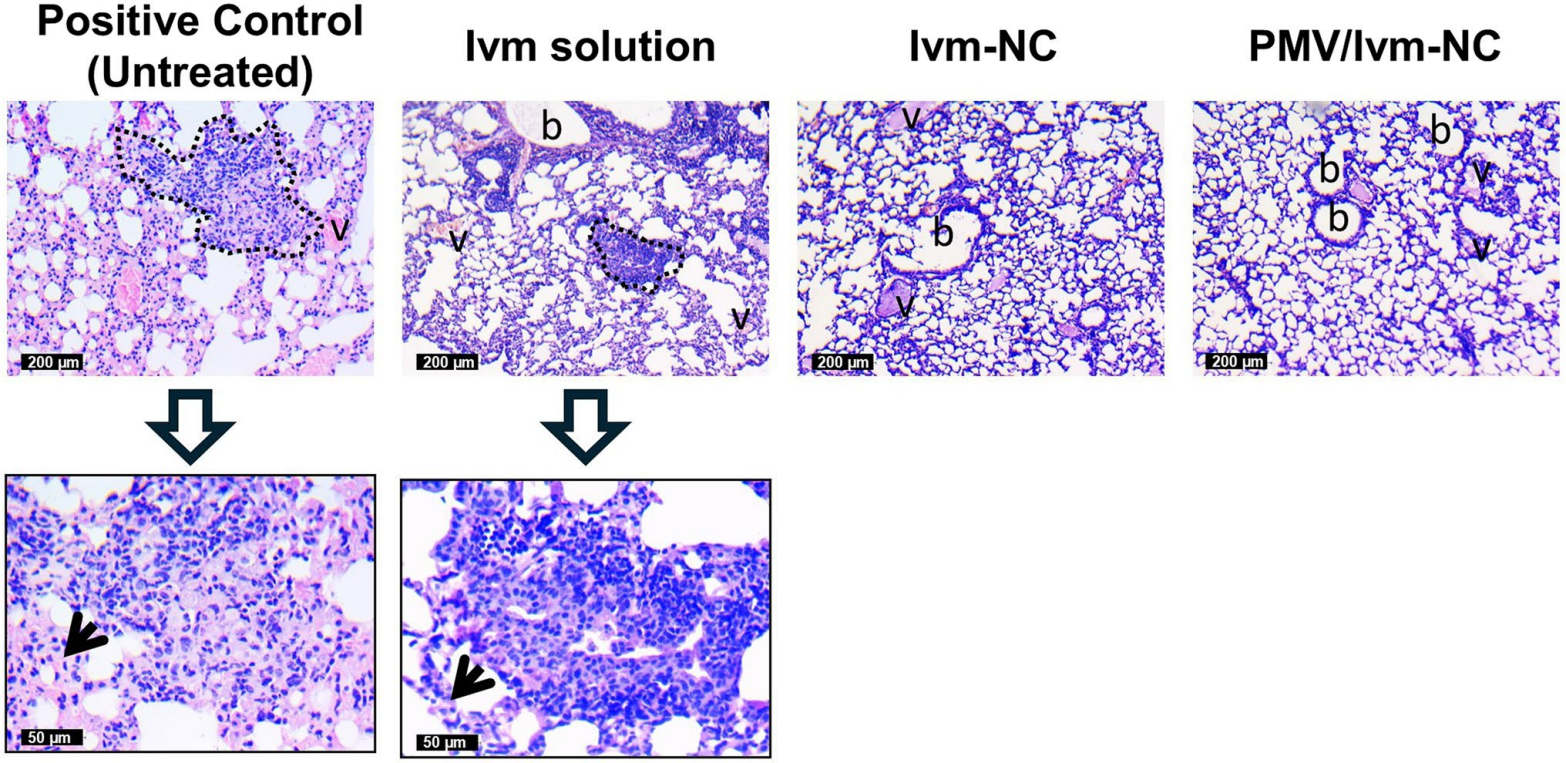
Assessment of metastatic nodules in H&E-stained lung sections of different treated groups. Lung sections are seen composed of alveoli with scattered bronchi (b) and vessels (v). Large metastatic nodules (dashed area) are seen in the positive control group. The inter alveolar septa are thickened and hypercellular (arrows). Metastatic nodules were still detected in Ivm treated group, however smaller (dashed area). Interalveolar septa were less cellular (arrows). High power view of metastatic nodule shows cohesive tumor cells. Meanwhile, no metastasis was seen in Ivm-NC and PMV/Ivm-NC treated groups. Upper panel: × 100, lower panel: high power (× 400)

Lung tissues of positive control (untreated) group showed metastatic tumor deposits seen as nodules of malignant cells Fig. 12. Metastatic nodules were seen mainly in perivascular zones and were large in size. They nearby lung tissue showed evident increased interstitial cellular infiltration. The inter alveolar septa were thickened with increased mononuclear cellularity. Metastatic nodules were still detected for Ivm solution group, however they were smaller and less frequent. The interstitium partially regained its normal thickness. Interestingly, Ivm-NC and PMV/Ivm-NC showed histologically free lung tissue with no sign of tumor metastasis, highlighting the enhanced antimetastatic efficacy of the nanoformulations.

Despite differences in experimental design and dosing regimen, our findings align with and complement previous reports on the antimetastatic potential of Ivm across diverse metastatic models. In a breast cancer model, Ivm combined with anti-PD-1 therapy produced superior metastatic inhibition compared to Ivm alone, as assessed by bioluminescent imaging and survival following primary tumor resection [97]. In a colorectal cancer model, intravenous injection of tumor cells, followed by intraperitoneal ivermectin administration similarly inhibited metastatic spread [77]. Taken together, these studies highlight the broad capacity of Ivm to interfere with metastatic progression across different cancer models. Importantly, our study demonstrates that nanocrystal formulation potentiates this effect in the triple negative breast cancer model, underscoring the potential of formulation strategies to maximize the drug inherent antimetastatic efficacy.

Given that platelets play a critical role in shielding circulating tumor cells (CTC) from immune clearance and promoting their survival in the bloodstream, platelet membrane coating was designed to leverage the platelet-tumor crosstalk via natural homing and adhesion properties of platelets to CTC and interfere with these interactions [100]. The equivalent performance between targeted and non-targeted nanocrystals indicates that, for Ivm, optimized drug delivery formulation may be the primary driver of its antimetastatic efficacy. Nonetheless, the role of the platelet membrane in facilitating interactions with CTC cannot be entirely disregarded and remains a plausible avenue for further investigation.

## Conclusion

In this study, we rationalized that the convergence of Ivm anticancer repurposing, nanocrystallization and platelet-mimetic camouflaging would offer a promising approach for comprehensive antitumor, immunotherapeutic and antimetastatic management of triple negative breast cancer. First, P188-stabilized Ivm-NC were successfully developed using a sonoprecipitation assisted anti-solvent precipitation method, to enhance Ivm solubility and its repurposed anticancer therapeutic functionality against triple negative breast cancer. Active targeting and tumor homing of Ivm-NC was endowed by biomimetic, platelet camouflaging of the developed NC with platelet membrane vesicles, exploiting the inherent tumor homing and immune evasion functionality of platelets. The resulting biomimetic PMV/Ivm-NC afforded superior in vitro selective cytotoxicity against the triple-negative MDA-MB-231 cells and normal cell cytocompatibility compared to PMV-free Ivm-NC. PMV functionalization secured higher cancer cell uptake and internalization compared to uncoated NC. In 4T1 tumor bearing BALB/c mice, the biomimetic PMV camouflage exhibited enhanced active targeted tumor homing with minimal off-target biodistribution. In addition, PMV/Ivm-NC exerted substantial antitumor efficacy with high tumor necrotic areas and low mitotic count, elaborated via multiple mechanisms, including induction of apoptosis, inhibition of angiogenesis and cell cycle progression. Notably, enhanced infiltration of CD4^+^ and CD8^+^ T cells within Ivm-treated tumors suggested the induction of immunogenic cell death, characterized by the activation of antitumor immune activation following cancer cell death. Finally, a promising antimetastatic activity was noted for PMV/Ivm-NC. These findings highlight the potential of combining drug repurposing, nanocrystal technology and platelet membrane camouflaging to create a multifaceted platform for triple negative breast cancer treatment.

Moving forward, optimization of preparation methods, particularly for platelet membrane isolation and coating, is required to improve yield and ensure reproducibility, which is critical for translational feasibility. In addition, detailed pharmacokinetic profiling in triple negative breast cancer animal models is essential to elucidate the in vivo behavior and systemic circulation time of the prepared formulation. Finally, a comprehensive evaluation of Ivm-induced immunogenic cell death including assessment of calreticulin exposure, and release of ATP and HMGB1 as classical markers of immunogenic cell death, would provide deeper insight into its contribution to the overall cytotoxic effects of the formulation.

## Supplementary Information

Below is the link to the electronic supplementary material.

Supplementary file1 (DOCX 1205 KB)

## Data Availability

The authors confirm that the data for this study findings are available upon request.

## Declarations

### Animal studies

All institutional and national guidelines for the care and use of laboratory animals were followed.
