# Supplementary material for: Biomimetic platelet-membrane camouflaged ivermectin nanocrystals for tumor homing and breast cancer management
Source: Drug Deliv Transl Res. 2026 Jan 6;16(8):2883–910. doi: 10.1007/s13346-025-02032-2 (PMC13346331; doi:10.1007/s13346-025-02032-2)
Supplement: Supplementary file 1 — Supplementary file1 (DOCX 1205 KB) [file 13346_2025_2032_MOESM1_ESM.docx]

Biomimetic platelet-membrane camouflaged ivermectin nanocrystals for tumor homing and breast cancer management

Supplementary material

**Marwa M. Sheir ^a^, Salma E. El-Habashy ^a, *^, Eman Sheta ^b^, Maha M. A. Nasra ^a^, Ossama Y. Abdallah ^a^**

^a^ *Department of Pharmaceutics, Faculty of Pharmacy, Alexandria University, Alexandria, Egypt.*

*^b^ Pathology Department, Faculty of Medicine, Alexandria University, Alexandria, Egypt*

*** ***Corresponding author****: Salma E. El-Habashy, Department of Pharmaceutics, Faculty of Pharmacy, Alexandria University, Alexandria, Egypt.*

*1 Khartoum Square, Azarita, Alexandria, Egypt. P.O. Box 21521, Alexandria, Egypt.*

***Tel:*** *+2 0109 193 5945*

***Email****:* [*salma.elhaleem@alexu.edu.eg*](mailto:salma.elhaleem@alexu.edu.eg)*,* [*dr.salma.essam@hotmail.com*](mailto:dr.salma.essam@hotmail.com)


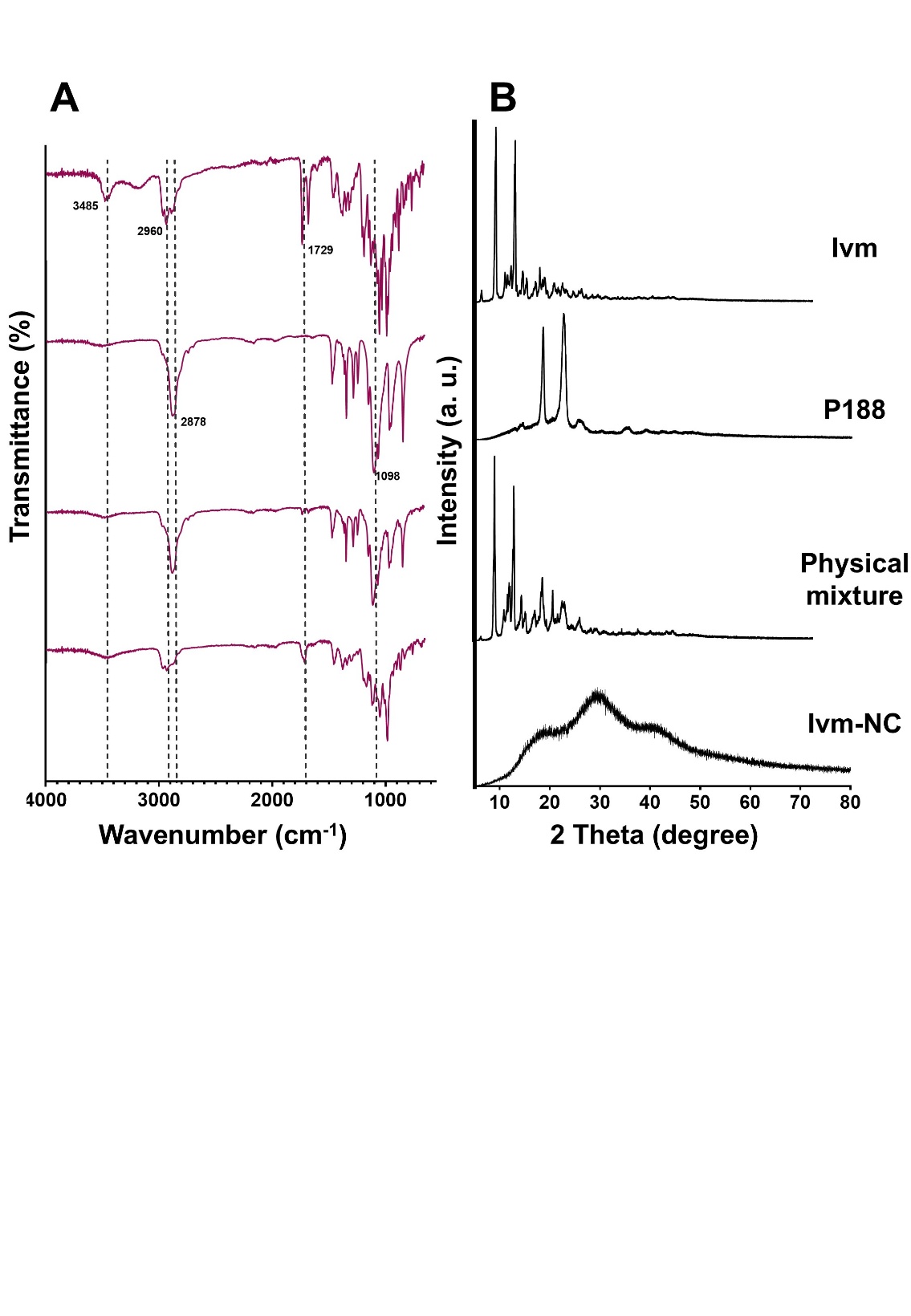


**Figure S1:** **Physicochemical characterization of the developed nanocrystals.** FTIR **(A)** and XRD**(B)**.


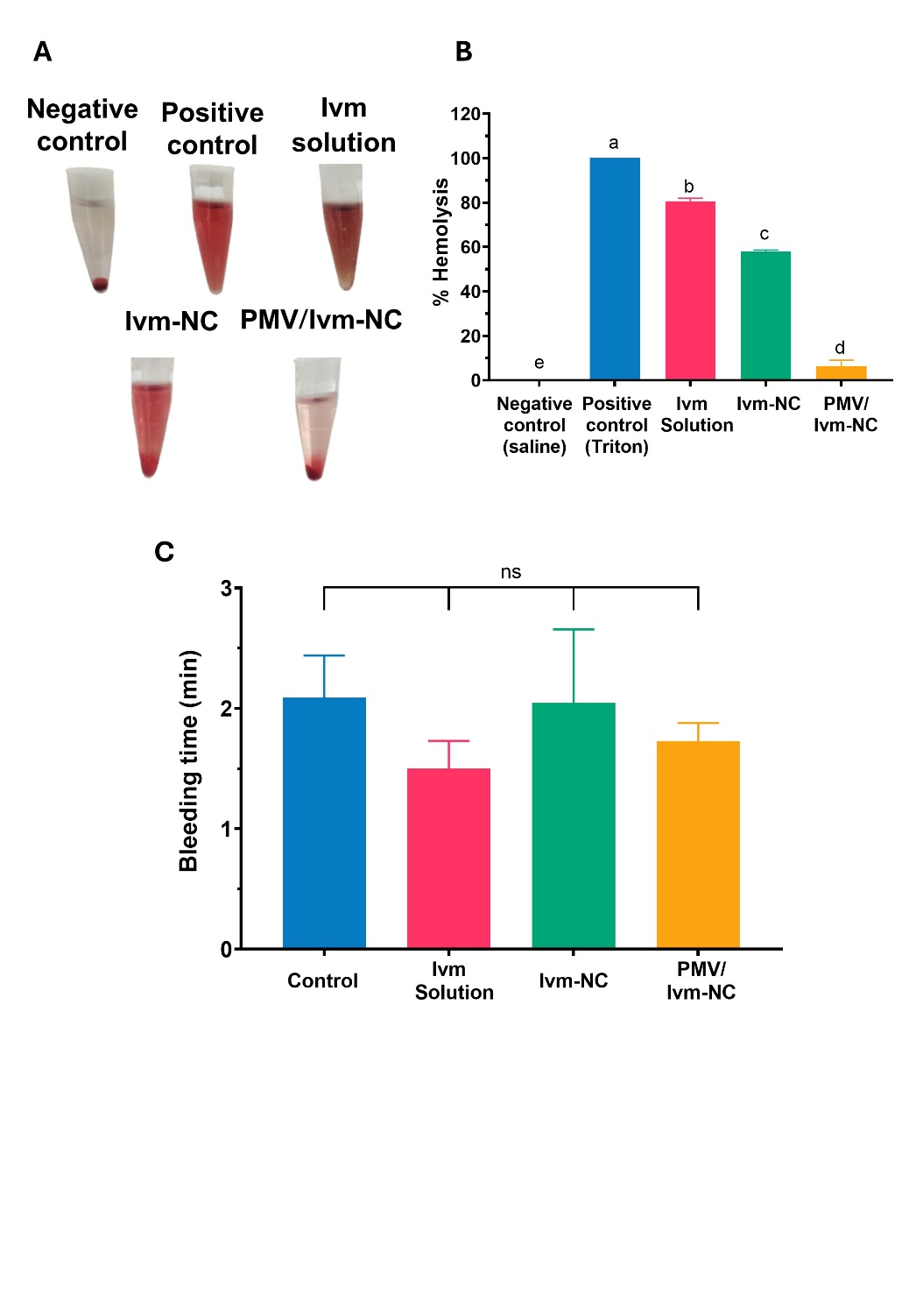


**Figure S2: Hemocompatibility evaluation of the developed nanocrystals (A-C).** Representative image of tests samples showing red blood cell lysis and hemoglobin release in the supernatant. Samples include negative control (saline), positive control (1% Triton X-100), Ivm-NC and PMV/Ivm-NC **(A).** Quantification of hemolysis expressed as percentage hemolysis relative to controls **(B).** Data represents mean ± SD, n = 3. In vivo tail bleeding time in mice following intravenous administration of the formulations, measured as the time (in minutes) until complete cessation of bleeding **(C).** Data represents mean ± SD, n = 3. Letters indicate statistically significant difference at *p* ≤ 0.05: a > b > c > d > e.

**
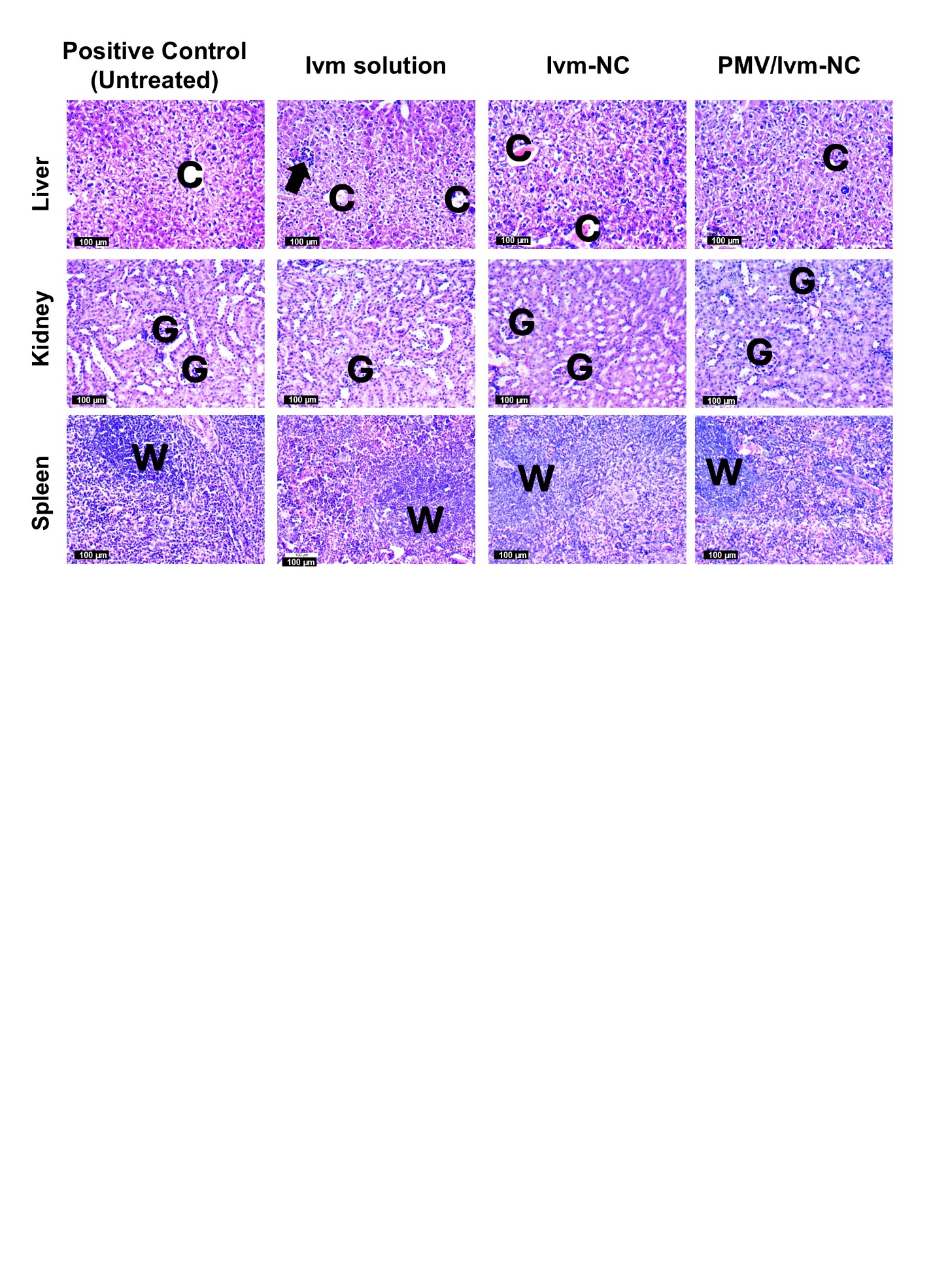
**

**Figure S3: Histopathological evaluation of major organs for systemic toxicity in mice of the in vivo study showing representative H&E-stained images (x200).** The upper panel represents liver tissue sections, where (C) represents central veins. In the positive control group, the liver showed normal architecture with no pathologic effect. In Ivm solution, lobular inflammation was seen (arrow). Meanwhile, Ivm-NC and PMV/Ivm-Nc groups showed normal architecture and histology of liver tissues. The middle panel represents renal tissue sections, where all groups exhibit evenly distributed glomeruli (G) within densely packed tubules. No pathologic changes are detected. The lower panel represents splenic tissue sections. White pulp lymphoid follicles (W) are seen within red pulp. No toxic effect is noted in different groups
